# Teaching Accurate Sex-related Biology: Approaches for More Socially Just Instruction

**DOI:** 10.1093/icb/icag136

**Published:** 2026-07-29

**Authors:** Sarah L Eddy, Jay J Falk, Megan G Massa, Karen M Warkentin, A M Aramati Casper

**Affiliations:** Department of Biology Teaching and Learning, University of Minnesota—Twin Cities, 5-220 Moos Tower, 515 Delaware Street SE, Minneapolis, MN 55455, USA; Center for Animal Behavior, Smithsonian Tropical Research Institute, Apartado Postal 0843-03092 Panamá, República de Panamá; Department of Ecology and Evolutionary Biology, Princeton University, 11 Ivy Lane, Princeton, NJ 08544, USA; Neuroscience and Behavioral Biology Program, Emory College of Arts and Sciences, Emory University, Atlanta,GA 30322, USA; Center for Animal Behavior, Smithsonian Tropical Research Institute, Apartado Postal 0843-03092 Panamá, República de Panamá; Department of Biology, Boston University, 5 Cummington Mall, Boston, MA 02215, USA; Biology Department and Graduate Degree Program in Ecology, Colorado State University, Fort Collins, CO 80523, USA

## Abstract

Current undergraduate biology education does not usually reflect what is known about the diversity of sex, reproduction, and sexual behavior across the diversity of life, nor does it commonly address the human-specific diversity of gender identity and sexual, romantic, and related orientations. Instead, research indicates many courses rely on oversimplifications and overgeneralizations of sex-related content, aligned with socially dominant narratives and norms. While these simplifications may seem like good starting points to facilitate learning, they can lead to multiple kinds of harm. These include creating confusion for students learning biology, limiting the questions biologists ask and how they interpret data, perpetuating scientific inaccuracies, and fostering public disbelief in more complex and accurate representations of sexual diversity. Moreover, for the increasing number of students whose lives do not align with these narratives, including those with queer genders and orientations, such oversimplifications in their courses can cause personal, interpersonal, and professional harm. In college, queer students are less likely to persist in STEM than their cisgender and heterosexual peers. In society, the broader impact of such oversimplifications gives credence to erroneous claims of “biological truth” being used to justify discriminatory laws and policies. Multiple groups of biology instructors, including some authors of this paper, have published recommendations for how to teach sex-related content in biology contexts more accurately and inclusively. However, putting these recommendations into practice can be challenging and there are few published examples of implementation. In this article we, a group of biology instructors and science education researchers who led a SICB 2026 education workshop on this topic, share how we have put these recommendations into practice in our own classrooms. We provide example activities, frameworks, and resources for readers to build from in their own teaching. Given that oversimplified biology is being invoked to strengthen harmful and discriminatory narratives in society, biologists have a heightened responsibility to teach contemporary and accurate sex-related content in their courses.

The variation in sex-related biology is amazing and our understanding of it is growing rapidly (Bachtrog et al. 2014; Jeffries et al. 2025), as highlighted in this special issue (McDonough-Goldstein et al. 2026; Warkentin et al. 2026). This variation includes diverse strategies of asexual and sexual reproduction, genetic recombination, sex determination, dimorphisms and polymorphisms that affect sexual compatibility, and discrete and continuous variation in gametic sex allocation (Billiard et al. 2026; Constable and Liu 2026; dos Santos et al. 2026; Falk 2026; McDonough-Goldstein et al. 2026; Van Etten and Johnson 2026; Warkentin et al. 2026). It includes variation in traits associated with sex allocation and reproductive strategies as well as diversity in life cycles and body plans that complicate our understanding of “sex” across multicellular lineages (Casper and Warkentin 2026; Chen and Pannell 2026; Constable and Liu 2026). It also includes diversity in sexual behavior and—in humans—orientations and identities (Lerch 2026). “Sex-related content” encompasses an expansive array of topics, including but well beyond “sex, gender, sexuality, and reproduction.” This special issue also highlights the rapid pace of scientific advances in understanding sex-related topics, ongoing challenges of grappling with this complexity and diversity, and variation in both conceptualization and language use. Sex-related content thus offers excellent opportunities to engage students in fascinating, cutting-edge science.

## The problem: Oversimplification of sex-related content is common in biology courses and causes multiple types of harms

The biological diversity of sex is often poorly represented in the classroom. Studies of biology textbooks across K-12, undergraduate, and graduate education found these texts only partially represent the diversity that exists in sex-related content. Instead, many texts center anthropocentric and androcentric perspectives on sex and reproduction, including reinforcing human gender roles even when writing about non-human organisms (Bazzul and Sykes 2011; Ray King et al. 2021; Spaulding and Fuselier 2023; Jackson et al. 2024). Many texts overemphasize mammals and birds which limits the reproductive strategies described (Ah-King 2013). Moreover, nonreproductive sexual behavior and sexes and genders beyond male/man and female/woman are rarely acknowledged (Snyder and Broadway 2004; Bazzul and Sykes 2011; Ah-King 2013; Ray King et al. 2021; Spaulding and Fuselier 2023). An analysis of common themes in sex-related content identified by students in college biology courses similarly found an emphasis on binary sex, heteronormativity, and sex and gender essentialism (Casper et al. 2022; Schneider et al. 2025).

The oversimplified and overgeneralized sex-related content being taught in biology courses is problematic from multiple standpoints. First, from a scientific perspective, it misrepresents organismal diversity and best practices for studying it. If the purpose of biology courses is to prepare students for biology-related careers (including health careers) then they should be learning current and emerging science. Oversimplified and overgeneralized content is, thus, a missed opportunity to educate. Second, oversimplification and generalization can create confusion and misconceptions that impact the advancement of science. This is illustrated by recent policy changes in the *Cell*, the *Lancet*, and Elsevier publishing, requiring researchers to specify how they determined the sex of their participants (Cell Press 2026). These changes were necessary because “sex” can be operationalized in many ways and ambiguity about how the term is used impacted the interpretation and comparability of studies (Goldman 2024; Eppley et al. 2026). Third, overgeneralization can hamper students’ understanding of large areas of biology. For example, many students are taught meiosis as part of gametogenesis in animals, so they learn that meiosis makes gametes. However, this is true only in organisms with diplontic life cycles (e.g., humans), yet this life cycle is often taught as universal (Casper and Warkentin 2026). In the many organisms such as plants, fungi, and algae with other types of life cycles, including haplontic and haplodiplontic, meiosis occurs at a different place in the lifecycle and *mitosis* makes gametes (Fusco and Minelli 2019). Thus, oversimplification both misrepresents most life forms and may predispose our students to have beliefs that make it harder for them to learn accurate biology. Therefore, to prepare students to be leaders in their future biology-related careers, we need to teach them the accurate and up-to-date science of sex-related topics.

A second class of harms are to students in biology courses whose lived experiences differ from the oversimplified sex-related content, including students who are intersex and/or have queer genders and orientations.^1^ A study with over 50 life science majors with queer genders in the United States, led by the first and last authors of this paper, identified a range of narratives about sex, gender, and reproduction in biology courses (Schneider et al. 2025). These narratives included binary sex, cisnormativity, gender essentialism, gender irrelevance, and compulsory sexuality among others. This study documented the ways such narratives impacted these students, from self-concept harms to harms to relationships and increased barriers in becoming competitive for biology careers (Schneider et al. 2025). For example, an intersex student shared multiple ways that the narrative of binary sex in their biology courses harmed them:

Many of my biology classes treat [the sexes] as if they’re two entirely separate planes. And it made me feel unwelcome , because it made me feel like I don’t exist. My existence directly contradicts the learning material, the exam material. And if I try to bring it up, I get penalized for it. I can’t just not answer the [exam] question or answer it intentionally wrong. But I know better than the question, because I lived it. And it’s made it really difficult for me. . . Because I felt like there wasn’t anything I could do. . . . I could tell that, over the years, it started to build up with a lot of self-loathing, wishing I was different or normal.

In this quote, the student described being erased by biology course content and the negative emotions that these experiences elicited. They also experienced a lack of agency to challenge these narratives, because writing biologically accurate responses that contrasted with the course content would lead to losing points on exams. Finally, they described how hearing that sex is binary repeatedly across their biology courses led to a reduction in their self-concept. Multiple stories like this one were also shared by students with queer genders, referencing multiple oversimplified narratives related to sex (Schneider et al. 2025). These experiences point to the importance of challenging oversimplified narratives to support the belonging and persistence students who are intersex and/or have queer genders and orientations in biology.

Finally, these oversimplified narratives contribute to societal harms for queer people broadly. Throughout history, biology has been used to reinforce what is considered normal (DuBois and Shattuck 2021; Massa 2026). When biology courses teach oversimplified content that reinforces gender essentialism, heteronormativity, and cisnormativity, those ideas appear to have the backing of science, which further entrenches their power in society. For example, sex as defined by the gametes an organism produces is being touted as “biological truth” and being enforced by federal and state governments to restrict the rights of people who are intersex and/or have queer genders and even to restrict what can be taught in college classrooms (Eddy and Long 2023; Exec. Order No. 14168 2025; Whitford 2025; Massa 2026). Yet, the reality in biology is that there are numerous concepts and operationalizations of “sex” (McLaughlin et al. 2023; Smiley et al. 2024; Eppley et al. 2026; McDonough-Goldstein et al. 2026; Warkentin et al. 2026). Given that the misrepresentation of biology is being invoked to strengthen these harmful narratives in society, biologists have a heightened responsibility to teach contemporary and accurate sex-related content even as the cultural context makes this more risky (Choi et al. 2025).

## What can biology instructors do?

The authors on this paper are all biology instructors who have sought out resources and expertise in sex-related areas of biology to use in our own teaching. We approach these topics from a range of disciplines and with different levels of experience and safety at our institutions. Collectively, we have training in neuroscience, evolution, integrative biology, and ecology. Some of us also have cross-disciplinary training in fields such as biology education and women and gender studies. Our teaching experience ranges from just a few years to over 25. Some of us are in tenured positions which grant relative safety, some are pre-tenure or non-tenure track, and some are in soft-money funded positions with no guarantee of long-term employment. We teach at both private and public R1 institutions. This paper builds from our Education Council sponsored TAL-X workshop at the 2026 Society for Integrative and Comparative Biology conference.

In our experience, there is not an extensive repository nor an easily accessible set of resources to support college level teaching of sex-related content in accurate and inclusive ways (although there are some resources at the K-12 level, see Table 1). Many of us have built our knowledge of these areas independently, spending great effort to track down articles in biology and interdisciplinary critiques and commentaries on biology, creating and revising teaching materials, and building our own teaching frameworks. We are writing this article to try to help lift that burden, at least a little, for others. Given the multiple harms resulting from oversimplified content, we and others recommend instructors teach accurate and up-to-date sex-related content and teach this content in inclusive ways.

**Table 1:** Summary of published recommendations by biology instructors for teaching sex, gender, sexual behavior, orientation, and reproduction related content

Overlapping recommendations related to biology content from the literature	Long et al. (2021)	Zemenick et al. (2022)	Casper et al. (2025)	Eddy et al. (2026)
1. Feature sex-related diversity across and within organisms to normalize variation. Integrate this diversity fully into units and across the course.	√	√	√	√
2. Avoid conflating sex and gender and using unnecessarily gendered language. Be consistent in language use across units.	√	√	√	√
3. Incorporate how social context/norms influences research and how biological data is interpreted through a human lens.	√	√	√	√
4. Have students draw their own conclusions by using constructivist teaching methods.	√			√
5. Bring discussions of gender into biology classrooms to avoid undermining its legitimacy. Consider teaching the entanglement of sex and gender.	√			√
6. Use both human and non-human examples to help normalize human diversity.		√		√
7. Present science knowledge as constantly under revision and still growing.		√	√	
**Resources for biology curriculum related to these recommendations:** https://www.genderinclusivebiology.com/ https://projectbiodiversify.org/ https://stemcenter.siue.edu/inclusive-biology-curriculum-impacts-on-students/ https://www.betterbiologynetwork.org/ The examples in this article. **Research on inclusive biology curriculum related to these recommendations**: Adams et al. (2026) Discussions of sexual diversity in nature increase student sense of belonging in biology. https://doi.org/10.1093/biosci/biag022 Blake et al. (2026) Inclusive biology curriculum interventions can reduce high school students’ bioessentialist beliefs. https://doi.org/10.1187/cbe.25-09-0207 Eddy et al. (2026) “There is a place for us here”: Exploring narratives supporting students with queer genders in biology courses. https://doi.org/10.1002/tea.70043				

*Note:* Check marks indicate that a version of this recommendation is included in the publication.

The first step in updating content is often updating our own knowledge of the state of a field. This is especially the case with sex-related topics, as current biological knowledge has not been integrated into textbooks. Browsing this special issue is one way to update one’s knowledge of these topics. This issue is full of articles ranging from richly cited overviews of the broad range of sexual biology and challenges in communication (Casper 2026; Casper and Warkentin 2026; Massa 2026; McDonough-Goldstein et al. 2026; Warkentin et al. 2026), to focused discussions of organismal groups (dos Santos et al. 2026; Van Etten and Johnson 2026), to new perspectives on key sex-related topics (Billiard et al. 2026; Constable and Liu 2026; Falk 2026; Lerch 2026). Another resource for catching up on content can be found in the supplemental materials of Casper et al. (2022) which includes an annotated bibliography of articles, book chapters, and books on the diversity of sex, gender, and sexual behavior in nature. In the supplemental materials of this article, authors Warkentin and Massa share readings they assign in their courses (see Supplemental Materials Parts C and E).

Multiple groups of biology teachers at the K-12 and college levels have offered recommendations for how to make sex-related content in biology more inclusive based on their experiences in teaching (Hales 2020; Long et al. 2021; Casper et al. 2022; Zemenick et al. 2022; Sharpe et al. 2023; Harris et al. 2024). Many of their ideas specifically for biology content overlap (see Table 1). One of the cross-cutting recommendations is to teach the diversity across and within organisms in sex, gender, and reproduction first before focusing in on one or a few examples. A second is to think carefully about the language we use in discussing sex-related topics. For example, when instructors just talk about sex in the biology classroom, without acknowledging gender, some students assume the instructor is conflating sex and gender (Casper et al. 2022). In addition, using inclusive language in one unit, but not consistently across all units, can undermine the impact of that language for students (Eddy et al. 2026). A final cross-cutting theme is explicitly acknowledging and exploring the roles of culture and how biology is socially constructed. This could take many forms, from teaching the history of scientific ideas to assigning students readings from critical and/or feminist scholars. Engaging in cultural discussions and with students’ prior knowledge are both important, as simply teaching the biological diversity of sex-related topics is mixed in its effectiveness (Casper and Dockendorf 2023; Casper, Hylton, et al. 2026).

In addition to recommendations, biology education researchers have been collecting data on the impact of sex and gender narratives in the classroom. For example, Adams et al. (2026) found that teaching the diversity of sex, gender, and reproduction across organisms and within humans increased the sense of inclusion of students with queer genders and/or orientations. Eddy et al. (2026) interviewed students who are intersex and/or have queer genders across the country and identified three broad messages in biology courses that increased their sense of belonging: (1) sex and gender are not the same; (2) sexes beyond male and female, genders beyond cis woman and cis man, and sexual behaviors and orientations beyond heterosexuality are legitimate; and (3) concepts of sex, gender, and orientation are influenced by both biological observations and societal norms. These three positive themes can manifest in the content through disclaimers, human examples, or non-human examples. Eddy et al. (2026) offers recommendations based on these interviews (see Table 1). They found these themes were more effective when more time was spent on them in class, biological evidence was provided (instead of relying on instructor authority alone), the content related to the themes was well integrated into the unit, themes were consistently used across the course, and content was presented with empathy.

Finally, trans and feminist scholars also offer recommendations for creating learning environments that integrate trans experiences and perspectives (referred to as critical trans education; Keenan 2017; Kean 2021; Martino and Omercajic 2021; Nicolazzo 2021; Hytten and Salas-SantaCruz 2016). Although diverse in specific approaches, they are united in calling on instructors to challenge binary, cisnormative, and essentialist thinking in our students and ourselves. They also highlight the need to avoid tokenism as we teach sex-related content focused on humans. Tokenism can occur through one-time lessons that make the diversity presented seem like an aberration rather than a normal part of variation or through presenting intersex or trans people in a way that makes them objects to be studied rather than people (Malatino 2019; Martino and Omercajic 2021; Rende Mendoza and Johnson 2024). One way to avoid tokenizing is to normalize biological diversity through integrating it across all topics, not just in sex-related content. A way to avoid specifically tokenizing human diversity is to teach diversity in both human and non-humans. Lane (2009) explains why this approach is valuable: “the complex human expression of sexuality and gender loses its uniqueness when situated in the extraordinarily diverse animal expression of sexuality and gender (pg. 142).” Finally, these scholars also call for representing the diversity of queer people across multiple dimensions in educational contexts, including their experiences of gender, race, and class (Driskill 2010; Martino and Omercajic 2021) as exclusion is not experienced equally by all queer people.

Recommendations from all these sources can be combined to design accurate, just, and engaging sex-related content for biology classrooms. Below, we share some examples of our approaches to teaching this content in college biology courses. All of these activities engage with multiple of the above suggestions from the literature. None of these activities or approaches have been formally tested through education research for effectiveness. We provide them as starting points: none are necessarily perfect as they are, but they offer something to build from in one’s teaching.

## Example activities that engage with accurate and just biology

### Sarah: Constructivist activities about definitions of sex

Constructivism is an approach to instruction where students interpret instructional activities involving new knowledge through the lens of their existing knowledge. It is a chance for them to connect new ideas to their existing knowledge and revise and/or expand what they know (Smith et al. 1993). Although not synonymous, the potentially more familiar approach of active learning often involves constructivist approaches. One of the advantages of constructivism can be that when students discover something for themselves, they are less resistant to it than when they are told to believe something (Casper, Bhaskar et al. 2026).

Here, I share two activities I have developed that support students to explore the multiple definitions of sex used by scientists using constructivist approaches (see Supplemental Materials A and B). The primary learning goal for both is to understand that sex is challenging to cleanly define and is an active space of research and scientific debate. This emphasis contrasts with the narrative students may have received from popular sources such as politicians, the media, and even possibly past biology courses. Thus, both activities align with Recommendations 1, 3, 4, and 7 (Table 1).

The first activity was developed for an upper-division course where students regularly read primary literature. In this activity, students read at least sections of three different recent papers that wrestle with how to define sex (for a total of 12 pages) ahead of class. One paper advocates for a gametic definition of sex, while the other two advocate for more complex definitions using multiple traits. Students create strength and weaknesses lists for the two types of definitions as well as consider the purposes for which the different definitions were developed (i.e., the gamete definition of sex has been used by evolutionary biologists to describe average strategies in a population, not to assign sex to every individual in a population). One benefit of this activity is that it features scientists, other than me, acknowledging the complexity of defining sex so it highlights that even experts are thinking about this and that this area is an active area of debate. It also provides space for students to draw their own conclusions. As a class, we make the strength and weakness lists, but I do not tell them what to decide. I assess the learning outcomes through a written assignment where they create a science brief for state officials who are considering legalizing a definition of sex for state licenses. In the brief, they summarize the “state of the field” and give their advice as a scientist about what definition to use (if any at all).

The second activity is one I have used when doing outreach with high school students. It does not require any student preparation ahead of time. In this activity, students are provided four case studies of animals with diverse sexual systems: the White-throated Sparrow (*Zonotrichia albicollis*), Iberian Mole (*Talpa occidentalis*), Clownfish (subfamily Amphiprioninae), and Japanese Common Grass Yellow Butterfly (*Eurema mandarina*). The primary learning goal for this activity is to recognize why determining a criterion to identify sex using any one trait, even just across animals, is challenging. Here, I use a broad characterization of sex as a classification system used in science to help researchers understand who can successfully reproduce with who. The student’s tasks are to read the four one-page case studies and complete a provided table to identify whether a series of traits that are popularly used to identify sex can reliably be used to identify sex in each species. Students then look across all the species to see if any one trait works consistently and, if not, consider how scientists should define sex. Finally, I debrief the activity using similar ideas to the first activity: that there is active debate in the scientific community about how to define sex. Again, this activity is a chance for students to wrestle with the complexity of sex. I like this activity because students get excited about the examples (many of which they have not heard of before). There are many additional organisms that could be used and the activity could definitely be expanded beyond animals. Because this was an outreach activity, there was no assessment, but I could imagine asking students to write a 3–5 sentence essay about what trait(s) they would focus on if they had to define sex and why.

In my experience, both activities have elicited high engagement from students and led to lots of new questions and curiosity. They have not been formally evaluated for their effectiveness and likely could benefit from modifications from readers based on their experiences teaching these topics. For example, instructors who were part of the TAL-X workshop suggested alternative readings (included in the supplements) that could be used for the definitions activity.

### Megan: Taking an interdisciplinary approach to sex in biology courses

An interdisciplinary approach can assist in teaching accurate and socially just sex-related biology content. Because scientific training and practice promotes the idea of the scientific process as objective and value-neutral (Whitt 2009), our positionality as scientists makes it difficult to take a critical reflexive view of our epistemology (Haraway 1988). By drawing on fields outside of the sciences (e.g., feminist and queer studies, science and technology studies, philosophy, and Indigenous studies), we can more readily unearth assumptions in our own foundational paradigms and move toward a science that not only centers the humanity of our practice but is also cognizant of its societal impacts.

I teach a two-course neuroscience series that promotes an all-encompassing view of sex through multiple lenses (for an annotated bibliography of readings and resources, please see Supplemental Materials part C). In the first course, Intro to Feminist Neuroscience, students examine neuroscience and biology from an intersectional feminist perspective. We explore how both sex and gender are racialized and collapse the sex-gender divide to understand how these two concepts co-create each other. Importantly, the syllabus also highlights intersex and trans identities and lived experiences. The course is designed to consistently encourage students to consider how social contexts and norms influence research, how our biological data is interpreted through a human lens, and how scientific knowledge is consistently changing (recommendations 3 and 7, Table 1). The class as a whole consistently challenges students to think beyond binary essentialist thinking.

I chose a historical entry point to demonstrate to students how social context/norms influence the science of sex. Students use this history to form their own opinions of the constructed nature of the biological framework of sex (Clune-Taylor 2021; Velocci 2024; Staller and Koerner 2025). As such, the introduction of sex variability is no longer a surprise but is instead evidence of more data that has been molded to fit into the current schema of sex and of more people that have been negatively affected by these schemas. Students first read key papers (highlighted in Supplementary Materials C) which develop a timeline of historical sex, witnessing the change in Western scientific conceptions and applications of sex (and gender) across time. Of particular note is the deployment of sex (differences) as a colonial tool (see Massa 2026, this issue, for elaboration), the human impacts of this deployment, and of the circular logic between sex and gender as concepts.

Thus, students are ready to think deeply and humanely about the biologies and individuals falling outside the norms of the sex binary with a relatively simple single-day lecture and activity (Supplemental Materials part D). In this activity, immediately after I provide a brief lecture on sex determination and sexual differentiation in a rather binary fashion, students apply this knowledge in ways that shatter the binary formation through the prediction of phenotypes of intersex traits. This process allows me to present the material in a straightforward way but not allow the learning to be codified in strictly binary bins. And by asking students to center personal experience of intersex people in their summative assessment, students practice humanizing biology. This destabilizing of the sex binary is also further elaborated later in the unit when I introduce students to the idea of embodiment of gender and entanglement of sex and gender (recommendation 5, Table 1).

After two units exploring gender impacts on scientific schemas, reviewing data undermining gendered assumptions, and having students apply their own critical lens to primary data articles (recommendation 4, Table 1), the final unit of the class centers the personhood and materialities of queer and trans individuals (see papers in Supplemental Materials Part C “Effects of the sex binary on marginalized populations”). When using human examples in class, we run the risk of minority communities turning into tools to be used as opposed to individuals and populations with their own distinct wants and needs (Rothbauer 2019). To combat this, I elevate the voices of affected members of the community within the frame of the impacts of science. For instance, intersex issues surrounding advocacy and classification are explored in the context of medicalization and norms established by the sex binary (Davis et al. 2016; Pagonis 2023; Semenya 2023; Weigel 2023). These are placed in direct contrast to trans issues and advocacy, for which the effects of science are equal and opposite despite similar etiologies via medicalization and binary sex norms (Spade 2003; Clune‐Taylor 2019).

While this course was constructed to be fully interdisciplinary, the strategies held within can be modified, scaled, and incorporated into any biology context, even down to a single portable activity (see Intersex Chart, Supplementary Materials part D). I encourage educators who have never attempted this type of interdisciplinary dialogue to start small and build out. Dropping consistent breadcrumbs throughout the course, making a theme instead of a one-off, builds trust and demonstrates conscientiousness. With this, educators and students alike build out their critical thinking capacities to take a look at the biology of sex from new perspectives.

### Aramati: Addressing the challenges of integrating complexity—Meiosis and life cycles example

Teaching the variation and complexity in sex-related topics (and others), particularly in introductory biology courses, is complicated by multiple structural barriers. As discussed in the introduction (above), these barriers include the need for each instructor to do their own learning and build their own course materials and lesson plans as well as the oversimplified knowledge that students often enter a course with (Casper et al. 2025). Therefore, even with diversity-centered content about sex-related topics, students can struggle to make sense of seeming conflicts between their prior knowledge and the complexity and variation being taught (Casper, Hylton et al. 2026). Additionally, while changing one or two activities can be a great starting point, it is eventually vital to integrate complexity throughout an entire course for holistic change (Table 1, recommendation 1).

To support student learning I employ two pedagogical strategies: cognitive bridging and identity threat protection (Kahan 2017; Vidak et al. 2019; Aleknavičiūtė et al. 2023; Aini et al. 2024). In combination, these strategies help students to connect new knowledge to their existing knowledge and can help students navigate threats that they may feel to their identities in this process. These threats may be related to either specific belief systems, such as a religion, or to their general sense of identity as belonging in science.

Cognitive bridging is a pedagogical strategy that aligns with constructivism and explicitly connects a students’ existing knowledge to new knowledge (Smith et al. 1993; Vidak et al. 2019). This contrasts with cognitive conflict, in which an instructor focuses on the conflict between a students’ pre-existing knowledge and what is being taught in the course—the goal, in cognitive conflict, is to cause the student to be dissatisfied with the prior knowledge, to motivate a shift (Posner et al. 1982; Aleknavičiūtė et al. 2023). However, from my experiences teaching in the current socio-cultural climate, cognitive conflict strategies may simply lead students to distrust the material being taught, or the instructor, instead of becoming dissatisfied with their existing knowledge. Cognitive bridging can help avoid this type of conflict, by creating space for the students’ existing knowledge and the new information being taught. Below, I describe how I use cognitive bridging to teach about life cycles in my introductory biology course.

Identity threat protection is a pedagogical strategy that can help support students’ emotional safety and sense of identity, to minimize internal conflict. Some students hold beliefs that may feel at odds with material being taught in a biology course. Much of the literature on identity threat protection is focused on how some Christian students may feel that learning about evolution conflicts with their beliefs (Aini et al. 2024, 2025). However, these strategies are likely to be applicable more broadly. One identity threat protection strategy is to normalize the process of learning new things in science, and that it is an important part of the practice of science to change how one thinks about the world as one gains new information (Barnes and Brownell 2017); this strategy is likely to support all students, since it may help support their sense of belonging in science and identity as they navigate learning that things they thought were true are incorrect. One easily implemented identity threat protection strategy I use is to write assessment questions that explicitly ask students to describe what was taught in this course. This question format can decrease internal conflict for students because I am not asking them to report what they believe, but rather to describe what was presented. It can also decrease the potential for a student to claim that an answer should be graded differently than it was (Table 1, Recommendations 4 and 7).

I use both cognitive bridging and identity threat protection in both sections of the introductory biology course I teach: One is a large (200+) student in-person lecture section, and the other is a small (~25 students) asynchronous section. Both sections include students with a wide range of backgrounds in science. In both contexts, many of my students find it challenging to learn about complex topics without clear boundaries, including many topics related to sex and reproduction. While I have used cognitive conflict strategies in the past, I have found cognitive bridging strategies to be more effective in supporting student learning. In my course, I have developed an arc of content about sex, reproduction, and related topics that starts with the basics of cells and cell function. In this process, I point to where we are going: We need to understand basic cell processes to understand the process of cellular replication, to understand how organisms reproduce. To prepare students to learn about life cycles, I teach students about basic cell components, chromosomes, different ploidy levels in different types of organisms, and the processes of mitosis, meiosis, and syngamy. I lay the groundwork for future learning through focusing on how meiosis creates new nuclei with half the ploidy of the original nuclei. I discuss that cytokinesis often happens with mitosis and meiosis to make new cells, but that cytokinesis is a separate process from both mitosis and meiosis. I also emphasize that the nuclei produced in meiosis and mitosis do different things in different types of organisms. In revising this basic content, I have had to focus on finding the implicit ways simplified vertebrate-centric life cycles are often embedded in materials for teaching this content, such as discussing that mitosis and meiosis make new cells (assuming cytokinesis is inherently part of these processes, i.e., ignoring syncytia), or that meiosis makes gametes. When the course materials I use make these assumptions, I either point the assumptions out to students, or ask them to identify the simplifications of the given process that are embedded in the materials. For example, I may ask students how they can tell that a meiosis video is implicitly assuming a specific life cycle type if it states that meiosis makes gametes. Identifying the implicit assumptions in different readings or videos I assign and in my own materials is an ongoing process; points of student confusion often help me to notice implicit assumptions I had previously missed. This semester, I have started using Perusall to provide some annotations of my own, and to facilitate asynchronous discussions of readings.

**Fig. 1:**
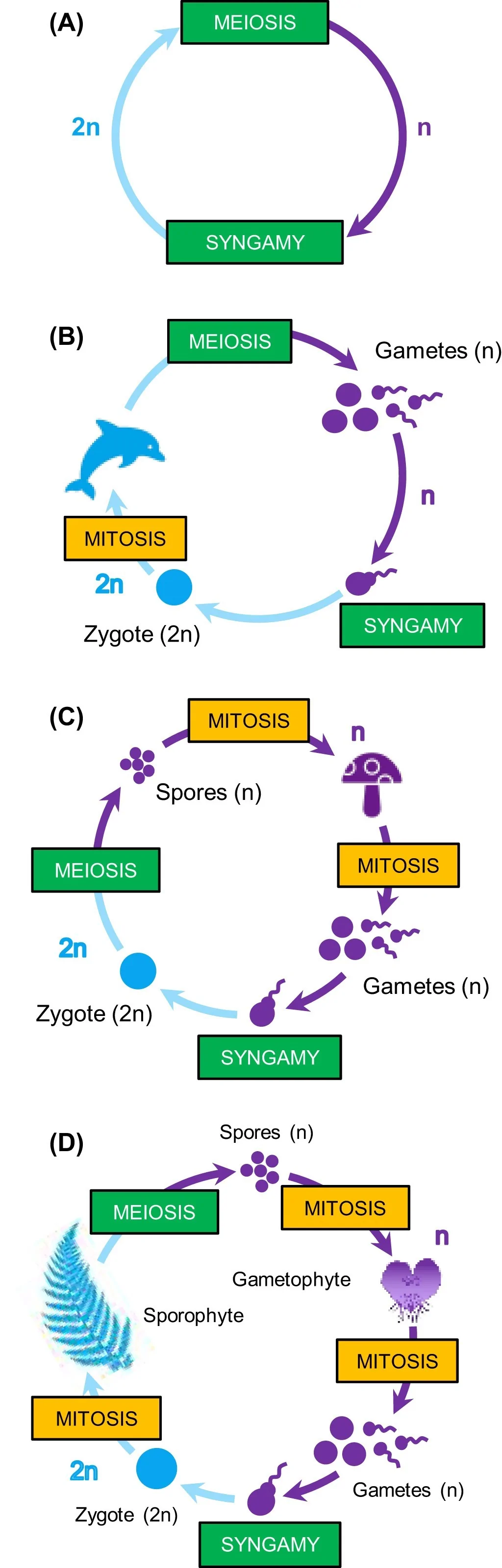
(A) A simple life cycle diagram, showing the basic movement between haploid and diploid stages through the process of meiosis and syngamy. Where mitosis happens in relation to meiosis and syngamy varies in the three common multicellular eukaryotic lifecycles. In diplontic lifecycles (B), only the diploid life stage goes through mitosis. Organisms with diplontic lifecycles include most animals and some algae and fungi. In haplontic life cycles (C), the spore goes through mitosis to become a multicellular organism, and that multicellular organism performs mitosis to create gametes. Organisms with haplontic life cycles include some types of fungi and algae. In haplodiplontic life cycles (D), the zygote and spore both perform mitosis to become multicellular organisms, and the multicellular organism that grows from the spore performs meiosis to make gametes. Organisms with haplodiplontic life cycles include all plants and some algae and fungi. The fern leaf is in the public domain; the mushroom is by difatama and the dolphin is by Moreno, both from the Noun Project; and the fern gametophyte is by author K.M.W.

To introduce life cycles, I start by stating that we are discussing common life cycles of multicellular eukaryotes. This situates the life cycles we discuss as only a subset of a greater range of diversity. We discuss that (typically) sexual reproduction in multicellular eukaryotes involves moving through meiosis and syngamy (Fig. 1A). Syngamy involves the merging of two gametes, changing the ploidy from n to 2n, and meiosis halves the ploidy of the resultant nuclei, going from 2n to n. Then, I discuss how different types of life cycles are characterized by when mitosis happens in relation to the processes of meiosis and syngamy.

After laying this foundation, I move through the three main multicellular eukaryotic life cycles. I start with a familiar life cycle—our own—and normalize that while students likely know the life cycle, they probably have not heard the name for it—“diplontic” (Fig. 1B). I expand from the basic life cycle diagram (Fig. 1A) to show how mitosis creates the multicellular diploid stage and meiosis creates gametes. Then, I bring in the haplontic life cycle, in which the multicellular stage is haploid, and the diploid stage is unicellular—the opposite of the diplontic cycle (Fig. 1C). We discuss how the diploid zygote goes through meiosis to create spores (reproductive cells that grow into a new individual, without combining with another reproductive cell), which grow into the multicellular haploid life stage. And, because it is already haploid, it has to perform mitosis to make gametes—it cannot perform meiosis because it is already haploid. Lastly, we discuss the haplodiplontic life cycle, which has both haploid and diploid multicellular stages (Fig. 1C). I start with gametes going through syngamy to create a zygote, which grows through mitosis into a multicellular organism, that then performs meiosis to make reproductive cells, and discuss how this is similar to the diplontic lifecycle. Then, I point out that this is where the haplodiplontic lifecycle diverges from the diplontic lifecycle, because meiosis makes spores, not gametes. These spores grow, through mitosis, into a multicellular haploid stage, which performs mitosis to make gametes (as in the haplontic lifecycle).

This scaffolding starts with the familiar and uses a comparative biology approach to help students create a cognitive framework for the complexity of the different life cycle types. Students who come in knowing that meiosis makes gametes have a space for that knowledge—meiosis does make gametes in the diplontic life cycle. However, they also gain information that goes beyond their existing knowledge through learning haplontic and haplodiplontic life cycles. This strategy demonstrates cognitive bridging because students are not wrong that meiosis makes gametes, they are simply learning that “meiosis makes gametes” is only applicable in some cases.

I continue to use this strategy throughout other topics in the course. Life cycles naturally build into discussions of the different concepts that the term “sex” is used for, as well as how different types of organisms have their gamete production arranged in different ways in different body plans. This semester, I am teaching with Warkentin et al.’s (2026) framework for different sex concepts, as part of my discussion of how there is no single agreed-upon concept of sex in biology. Rather, there are numerous sex concepts and the one that is useful in a given situation is context-dependent. I have found it helpful in being able to more easily articulate the many different ways the term sex is used in biology, and to implement Eppley et al.’s (2026) recommendations for simply naming how one is using sex in a given context.

The organismal component of the introductory course I teach is focused on plants, which inherently require a diversity-centric perspective about sex and reproduction. The language we use about plants also requires cognitive flexibility, as the term “gender” in plants describes a quantitative characteristics that either describes the proportion of each gamete type an individual produces (phenotypic gender), or the offspring an individual produces from its different gamete types (Casper 2026; functional gender; Lloyd 1980; Pannell 2023). In introducing the concept of quantitative gender, I discuss the socially constructed nature of terms (i.e., we assign meaning to words), and briefly discuss the differences between sex and gender in humans, then contrast it to how we use gender in plants (Table 1, recommendations 2 and 5).

Many of the plant-centric concepts, such as modular body organizations, quantitative gender, and the plethora of plant-specific language that exists to explain the organization of reproductive structures in plants can be, as Haufler et al. (2016) wrote, “baffling” for students. However, I repeatedly return to the concept they are introduced to via life cycles: Plants have a way of being that is very different from our own. Their life cycles, modularity, and reproductive processes (and all of their other ways of being) are not inherently unusual or difficult to understand. Instead, it is our human, mammalian, and vertebrate-centric perspectives and assumptions that situate us such that we think of plants as unusual or difficult (Table 1, recommendation 3; Casper and Warkentin 2026). If we were haplodiplontic modular organisms ourselves, then we might find diplontic unitary organisms baffling. Situating our human perspective within the landscape of biological diversity creates a cognitive bridge between our own biology and the biology of other organisms. I leverage the plant-centric focus of the course for identity threat protection: Humans (and all other animals) are beyond the scope of the course, and learning about the diversity of sex-related content in organisms so unlike ourselves may be less likely to trigger identity threat related to human gender identities and sexualities. Creating bridges between the familiar and new may also reduce the potential identity threat caused by the challenges of understanding biological complexity. Such challenges are a normal and inherent part of trying to grasp the vast diversity of biology as a vertebrate, mammal, and human.

### Karen: Elements of a “Diversity of Sex” course

To integrate teaching about diversity—including but not limited to diversity of sex—throughout a course, I take a multifaceted approach. I establish a conceptual foundation grounded in the mechanisms that generate variation. I use recent advances to emphasize the continual change in scientific knowledge and set the expectation that some things we think now will turn out to be wrong. I ask students to question assumptions and practice critical reading, reinforcing that they can critique published science. I also stress that work can be useful without being perfect, making room for individual and collective growth in understanding. Overall, I discuss science as a socially situated human process, with feedback loops that can limit and facilitate change. These approaches, together with the content I include (e.g., syllabus and readings in Supplement E), integrate all the pedagogical suggestions in Table 1. To illustrate these pedagogical approaches, below I share examples from my senior/graduate level “Diversity of Sex” biology course. I have also used similar approaches and topics in other classes, including for freshman non-majors and in an interdisciplinary team-taught class.

**Fig. 2:**
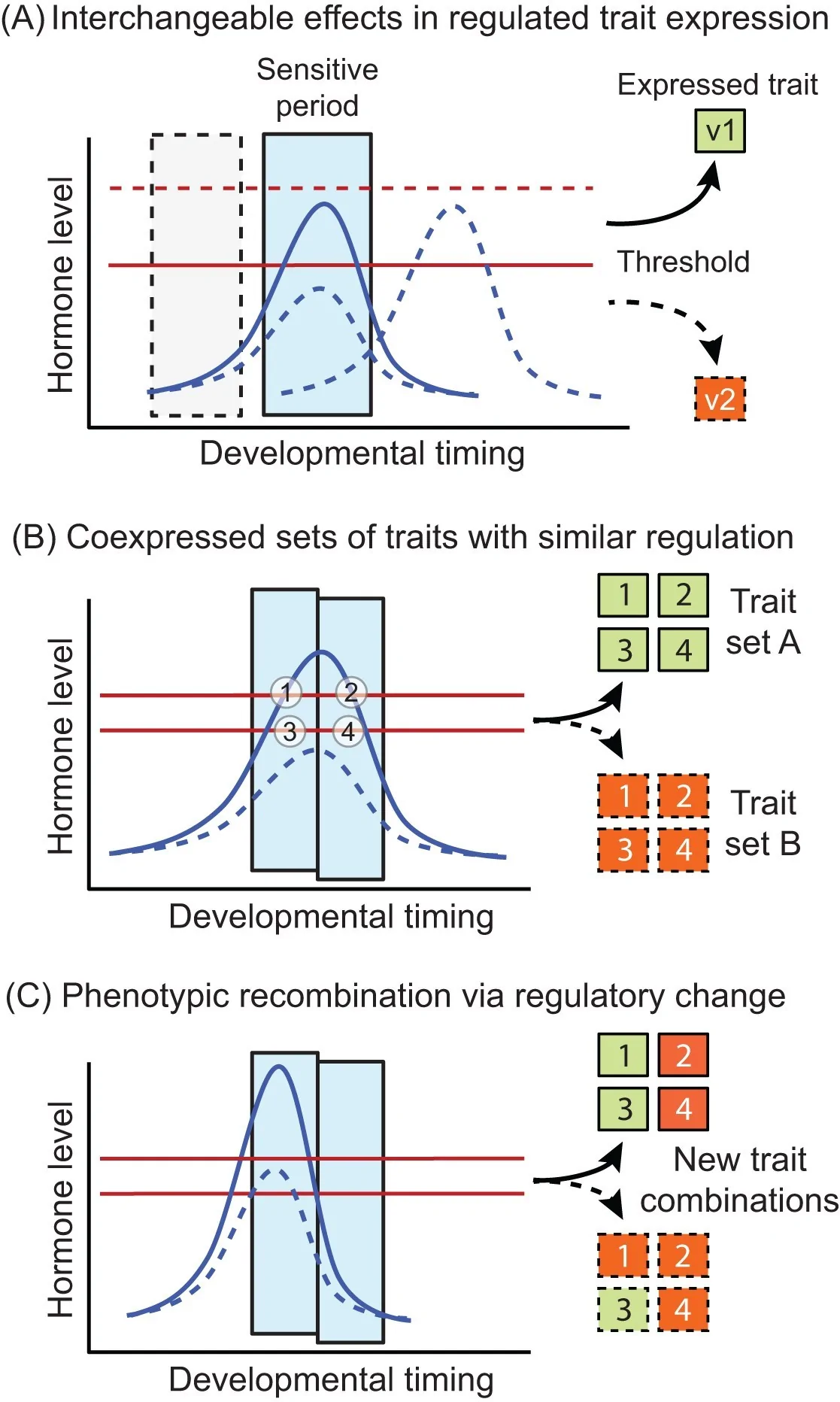
Multicomponent regulation and variation in trait expression. (A) If a hormone level exceeds a threshold during a sensitive period when its receptors are expressed in a tissue (solid lines), version 1 of a trait is expressed (black label); if not (dashed lines), version 2 is expressed (white label). A delay in hormone release, reduction in hormone level, earlier expression of receptors, or decreased receptor sensitivity all have the same effect on trait expression. (B) Four traits (1–4) are regulated by the same hormone, with slightly different thresholds and sensitive periods; traits 1 and 2 have a higher threshold, while traits 2 and 4 have a later sensitive period. These traits are coexpressed, forming alternative trait sets (A and B) characteristic of alternative morphs that express high (solid) or low (dashed) levels of the hormone during the adjacent sensitive periods. (C) A small regulatory shift from the scenario in panel B leads to faster hormone release in both morphs, increasing the level and shortening the duration of the hormone peak. Given the slight differences in regulation of each trait, this generates new phenotypic combinations.

To build a foundation for understanding diversity, I introduce phenotypic plasticity as a framework for integrating genetic and environmental effects. I discuss a core suite of developmental mechanisms that build bodies, including regulated gene expression, epigenetics and hormones, and how these mechanisms generate variation at multiple levels, from individuals to species, and enable evolution. To illustrate, we consider how trait expression is regulated by complex, multicomponent mechanisms. For instance, if sufficient levels of a hormone bind to receptors expressed in a tissue during a sensitive period, that can change the tissue’s gene expression pattern, thereby altering the expression of a downstream trait (Fig. 2A). Such mechanisms function as developmental switches. Because multiple genetic and environmental factors can alter components of the switch mechanism, they can have interchangeable effects on expression of the trait. Bodies are structured as modular, hierarchical groupings of traits whose regulatory coordination varies. Sets of traits can be regulated by a shared or similar mechanism, and thus commonly expressed together, as in alternative morphs or life stages (Fig. 2B). Nonetheless, relatively small genetic or environmental changes can generate new combinations of traits, including common variations and striking evolutionary novelties; this is called phenotypic recombination (Fig. 2C). Because sexes are developmental alternatives, based on differential expression of largely or entirely shared genomes, these developmental principles apply to variation between and within sexes. Thus, ancestrally male and female traits can be phenotypically recombined via cross-sexual transfer, generating individual variation, alternative ways to be male or female within species, and species differences; there are many examples of this kind of diversity (West-Eberhard 2003; Anderson and Falk 2023).

To normalize and model the process of individual growth and changing scientific knowledge, I often tell students how my—and our—understanding has changed. This is particularly relevant for topics where students enter a class with oversimplified, overgeneralized, or outdated knowledge. For instance, I tell them how I used to teach that vertebrates, generally, have obligately sexual reproduction, while noting the many origins of parthenogenetic all-female species. Then, reports of facultative parthenogenesis in sexually reproducing vertebrates started appearing—first for females in captivity without males (Watts et al. 2006), then in the wild (Booth et al. 2012), and then in captivity with males (Kratochvíl et al. 2020; Ryder et al. 2021). Recently, Kratochvíl and colleagues (2020) documented frequent, cryptic facultative parthenogenesis in a tropical night lizard. Single litters include fatherless and biparental offspring of both sexes; all offspring are fertile and females have both reproductive options, regardless of their own parentage. Such flexibility is difficult to detect, and we have rarely looked for it. I now try to be explicit about limitations to current knowledge and clear that absence of evidence is not evidence for absence, and I encourage students to consider what diversity we might currently be missing. I mentor students to do literature searches and share their findings to expand their classmates’ and my knowledge of the diversity of sex. Practically, we need to teach for the future with available resources, while both scientific and social progress continue to change our understanding and the literature includes a mixture of valuable information and outdated, flawed, or even offensive elements. I explicitly acknowledge this and use social annotation of texts (through Perusall) to build critical reading skills, enabling students to support each other in asking questions, cross-checking information, digging deeper, and identifying highlights and flaws in readings, which we explore further in classroom discussions. These elements help to build a collaborative, discovery-oriented community of sex diversity scholars.

The fact that science is a human, social process is embedded throughout my teaching on sex diversity. For instance, I have students read van Anders (2014) on “nomenclature and knowledge-culture” to introduce the epistemology of ignorance and consider how our language shapes questions, research, and knowledge. As a case study, illustrating how social expectations about sex roles affected biologists’ ideas about sex differences, including overgeneralizations from groundbreaking but flawed experiments that shaped and framed research for decades, students read Tang-Martínez (2016) on “rethinking Bateman’s principles.” Darwin’s sexual selection theory includes key insights and has motivated a rich body of research; however, it was also part of a mutually reinforcing cycle of social and scientific ideas about binary sex roles that slowed our appreciation of their diversity, which is now abundantly documented in nature (Ah-King 2022). I follow this critique by situating current research on sexual selection, in both sexes, within the broader context of social selection. Darwin recognized that competition for mates (or their gametes) can oppose selection by nonsocial ecological interactions, explaining the origin of extravagant traits that reduce survival. West-Eberhard explained how social competition for any resource—not just mates—can generate similar evolutionary dynamics; thus, sexual selection is part of the larger process of social selection, which can explain the evolution of ornaments and weapons in any sex and life stage (West-Eberhard 1983; Tobias et al. 2012). More generally, I encourage students to think about their own understanding and our collective knowledge in the context of historical and social factors, asking “Why didn’t I (or we) know that?”

These elements are part of an integrated strategy to normalize sexual diversity and complexity, contextualize our rapidly growing but still patchy understanding of this diversity, apply concepts and tools developed in other biodiversity contexts to sex, and use sexual diversity to teach fundamentals of development and evolution. The foundational conceptual framework of phenotypic plasticity, analysis of science as a social process, and community practices that support students as learners and empower them as scholars are all key elements of this endeavor. However, I did not begin by teaching an entire, integrated sex diversity course. I developed content knowledge and pedagogical strategies for this through years of reading and practice, trying them out bit by bit and learning with and from my students. I think there are many ways that pieces of this strategy and elements or modules of content could be used in courses with at least partially overlapping goals. Even small changes can make a difference for students and contribute to building instructor capacity (Casper et al. 2025).

### Jay: Teaching sexual diversity through a writer’s lens

Sex-related biology topics are addressed in a wide range of courses, including those for non-majors and disciplines beyond biology. These courses can provide flexible opportunities to introduce a wide range of students to the diversity of sex in biology. For students of many majors, topics and issues related to sex intersect personal identity and academic curiosity. The fascinating diversity of ways that organisms engage in reproduction can break commonly held assumptions and intuitions. Educators can use this to their advantage to integrate natural science with social science and history, allowing students from a variety of backgrounds to engage in topics that matter to their lived experiences. In Fall 2020, I designed and taught a remote course based on the biology, evolution, and diversity of sexes through a First-year Writing Seminar program, which allows instructors to provide writing instruction focused on specialized topics for mixed major students. My course design was motivated and inspired by my research with a species that expresses extensive sex-associated variation (Falk et al. 2021) and interactions with Karen Warkentin, in the field in Panama and as a guest lecturer in their Sex Diversity course. My course overlapped theirs in topics and ideals but was structured around the process of writing. A writing focus provides several benefits for teaching about the diversity of sexes, many of which I did not realize before teaching the course. Here, I briefly describe some advantages to making writing a central aspect of teaching the evolution of sexes and sexual diversity across taxa.

As noted above, many K-12 and college biology courses emphasize oversimplified content on reproduction, heteronormativity, and binary sex distinctions. Alternatively, confronting the complexity of biological diversity while acknowledging science as a socially embedded human practice may involve deeper more critical thought processes. Teaching students to engage in these novel topics by doing their own research offers them the chance to actively develop their own voice. It gives students more agency in their own learning process, avoiding the perception that they are being “forced” to adopt a particular perspective (Table 1, recommendation 4). An example of a writing assignment using this approach was a blog post assignment (Supplemental Materials Section F). For this assignment, students read one of several research papers I had compiled that engaged with diverse sex-related topics such as sex-switching species and parthenogenesis. Students were required to briefly summarize and also use their first-person voice to express their own opinions of the research, based on related papers that they found during independent study. This allowed for independent research with a guided starting point but also encouraged development of a personal voice, something that many students struggle with during their first years of post-secondary education.

Working with sexual diversity through a writer’s lens was also useful in confronting the science-society interface (Table 1, recommendation 3). By engaging with examples of both academic research papers and the news articles that covered them, students learned how scientific research can be portrayed in oversimplified ways when attempting to reach public audiences. We began by first reading “Accommodating Science: The Rhetorical Life of Scientific Facts” (Fahnestock 1993) which demonstrates the rhetorical shift that occurs when science is written for expert peers versus the public eye. My Science Communication assignment asked students to analyze an academic research paper and a news article about the findings, encouraging them to evaluate both works for their success in communicating the results and implications of the research (see Supplemental Materials Section F). Understanding rhetorical shifts, and the “accommodations” that occur when research moves between audiences is a powerful way of demonstrating how scientific discovery can interact with social narratives, especially when it comes to human sexual identities. Furthermore, this helps students develop science literacy and science communication skills.

There is a certain joy in knowing that we as humans (unitary, gonochoric, and diplontic organisms), occupy just a tiny portion of the range of life cycles and reproductive possibilities that exist on this planet. Writing can provide students an opportunity to engage in the cultural and social implications of such diverse possibilities in imaginative and creative ways. Modifying an assignment from Warkentin’s introductory non-majors course, I had students read two science fiction stories, by Octavia Butler and Ursula Le Guin (Butler 1984; Le Guin 2002), then asked them to imagine a society with a non-human form of reproductive biology and to write a short story based on this imagined world (see Supplemental Materials Section F). I also asked students to write an Afterword to reflect on their own writing, including how they shifted their voice from a focus on research to an imaginative process, as well as their inspirations and their identity as a writer, and by extension, a thinker. Imaginative and creative works are important sources of inspiration (e.g., Imbler 2022; Gumbs 2022) that are rarely credited in a traditional biology course.

Though incredibly rewarding, teaching this course was also scary. I had no formal postgraduate training in the topics I was teaching. When I assigned papers from the social sciences and humanities (see Supplemental Materials Section F), I worried that I was not doing the works justice. I read a lot and did a great deal of research on both the course topic and on methods for teaching writing to prepare myself. I wanted to teach a course that was, at the very least, not harmful to students with diverse identities and, if possible, inspiring to them. With that said, I made mistakes. As one example, I realized soon after starting the course that the title “Females, Males and all the In-betweens” was unfortunate, making it seem as if females and males are the ends of a spectrum and all other forms of sexual diversity fit between them. However, without teaching the course, I may not have found my way to that perspective, and I look forward to improving this course in a future iteration.

## Conclusion

We hope the examples above and in the supplemental materials provide readers with ideas for how they can incorporate accurate and just sex-related content into their own courses. As demonstrated here, there are a diversity of ways to attempt this. Some may work better in some courses and contexts than others. Some of these strategies (such as describing the diversity in non-human organisms) may appear more neutral and may be more enactable in places with heightened scrutiny on these topics. Across any context, engaging in constructivist, bridging, and identity threat reduction strategies may support student engagement and learning. In addition, teaching how scientific knowledge is continually growing and changing is helpful and accurate no matter the content being taught. We encourage our readers to try out some of these strategies. Teaching accurate and just sex-related content in biology courses is critical to prepare students to be leaders in biology-related careers, advance science, make biology welcoming to all communities, and counter the misuse of science in society.

## Supplementary Material

icag136_Supplemental_File

## Data Availability

Data sharing is not applicable to this article, as no new data were created or analyzed in this study.

## Footnotes

1 There is no widely agreed upon term to group individuals who identify with terms such as trans, nonbinary, gender non-conforming, and/or questioning their gender identities, particularly when trying to include Indigenous gender/sex/ualities (Picq and Tikuna 2019). When referencing humans, we have chosen to use the term queer people to describe all people with non-cisgender or non-heterosexual identities, the term people with queer genders to describe trans, non-binary, gender non-conforming, and questioning people, and the term people with queer orientations to describe those with non-heterosexual sexual, romantic, and related orientations.

## Notes

From the symposium “Sex across origins: Questioning animal-centric assumptions and developing integrative frameworks” presented at the annual meeting of the Society for Integrative and Comparative Biology, January 3–7th, 2026.
