# Supplementary material for: Teaching Accurate Sex-related Biology: Approaches for More Socially Just Instruction
Source: Integr Comp Biol. 2026 Jul 29;66:icag136. doi: 10.1093/icb/icag136 (PMC13555826; doi:10.1093/icb/icag136)
Supplement: Supplementary file 1 [file icag136_supplemental_file.pdf]

Supplemental Materials

**Section**	**Pages**	**Resource Type(s):**	**Focus of Course initially Developed for:**	**Level of Course Used in**
**A**	2 – 3	**Lesson Plan** for class period on the diversity of characterizations of sex used in biology.	Reading & discussion intensive biology course	Upper or lower division
**B**	4 – 8	**Lesson Plan** for an activity exploring the diversity of sex and sex determination in animals.	Outreach	High School or lower division
**C**	9 – 17	**Annotated Bibliography** of readings in science and technology studies with an emphasis on neuroscience.	Neuroscience, Science & Technology Studies	Upper division
**D**	18 – 20	**Lesson Plan** for teaching sex determination, sexual differentiation, and sexual diversity through human intersex examples	Neuroscience	Upper division
**E**	21 – 28	**Syllabus** and **Readings** for a Diversity of Sex Course.	Reading intensive biology course	Upper Division
**F**	29 – 33	**Lesson Plans** for teaching sex diversity through a series of writing assignments:<br>• Annotated Bibliography Blogpost (pgs. 29 – 30)<br>• Comparing and Contrasting Popular and Academic Writing Styles (pgs. 30 – 31)<br>• Speculative Fiction: Science as Storytelling (pgs. 31 – 33)	Writing intensive course	Lower division

A. Activity: Definitions of Sex

Creator: Sarah L. Eddy (seddy@umn.edu)

Timing: 60 minutes to 2 hours

Context Developed for:

- Upper division course
- Discussion-based course
- Reading and writing intensive course

Learning Goals for Activity:

- Explore the diversity of ways scientists are currently characterizing sex and how definitions of sex are not a settled topic in biology.
- Describe gamete-based, multi-modal, and multi-level models of sex.
- Understand the strengths, weaknesses, and constraints of each of these models.

Activity Components:

Assigned outside of class reading (12 pages total)

- Goymann, W., Brumm, H., & Kappeler, P. M. (2023). Biological sex is binary, even though there is a rainbow of sex roles: Denying biological sex is anthropocentric and promotes species chauvinism. Bioessays, 45(2), 2200173. https://doi.org/10.1002/bies.202200173 (**5 pages**)
- *Introductions to*:
    - Smiley, K. O., Munley, K. M., Aghi, K., Lipshutz, S. E., Patton, T. M., Pradhan, D. S., & Solomon-Lane, T. K. (2024). Sex diversity in the 21st century: Concepts, frameworks, and approaches for the future of neuroendocrinology. Hormones and behavior, 157, 105445. https://doi.org/10.1016/j.yhbeh.2023.105445 (**3 pages**)
    - McLaughlin, J. F., Brock, K. M., Gates, I., Pethkar, A., Piattoni, M., Rossi, A., & Lipshutz, S. E. (2023). Multivariate models of animal sex: Breaking binaries leads to a better understanding of ecology and evolution. Integrative and comparative biology, 63(4), 891-906. https://doi.org/10.1093/icb/icad027 (**3 pages**)

Additional Potential Papers recommended by workshop participants:

- Velocci, B. (2024). The history of sex research: Is "sex" a useful category? *Cell*, *187*(6), 1343-1346. https://doi.org/10.1016/j.cell.2024.02.001
- Pape, M., Miyagi, M., Ritz, S. A., Boulicault, M., Richardson, S. S., & Maney, D. L. (2024). Sex contextualism in laboratory research: Enhancing rigor and precision in the study of sex-related variables. *Cell*, *187*(6), 1316-1326. https://doi.org/10.1016/j.cell.2024.02.008

- Eppley, M. G., A.Lee, R.Dellinger, and A.Swank. 2026. “There is no consensus on biological sex.” Ecology Letters 29(3): e70350. https://doi-org.ezp3.lib.umn.edu/10.1111/ele.70350

In class discussion prompts:

1. How did each of these papers define sex (i.e., what biological variables were they using)? How many sexes resulted from each approach? What field are each coming out of?
2. What are Goymann et al.’s critiques of more body-based models of sex?
3. Goymann et al. also point out their own definition of sex has limits. What are those limitations?
4. For each of the remaining 3 papers (Smiley, McLaughlin, & Uyeda): What are the author’s critiques of the gamete model of sex?
5. For each of the remaining 3 papers (Smiley, McLaughlin, & Uyeda): What do the authors propose is a better way to think about sex & why?

Assessment:

For this assignment you will create an Instagram post with 8-10 slides (~450 words) that addresses the following prompt:

> “The state of Minnesota is considering writing a law legally defining sex. The governor's advisors ask you, a well-known biologist, to advise on how to write this law. They want to know the consensus in biology on what traits to use to define sex. Based on the readings in this unit, prepare an educational brief (in the form of an Instagram post) for law makers about what you think we can and cannot conclude from biology about how to define sex. As part of this brief include the pros and cons of using gametes alone and one other trait of the body (hormones, genitalia, etc) alone to define sex as well as your suggestion, based on the evidence, of how to move forward.”

B. Activity: Diversity of Sex in Animals

Creator: Sarah L. Eddy (seddy@umn.edu)

Timing: 30 minutes

Context Developed for:

- High school

Learning Goals for Activity:

- Identify the diversity of traits used to define sex in animals.
- Explore some of the diversity of sexual systems in animals.
- Recognize that no single trait can be used to reliably define sex across all these organisms.

Activity Components:

Introduction

- Slide 1: Sex is a classification system used in science to help researchers understand who can successfully reproduce with who. You may have heard different people talk about different traits that can be used to classify sex.
- Slide 2: Activity 1 (in pairs): What traits have you been told can be used to classify sex in biology classes (i.e., what feature determines sex)? And/Or: What definitions of sex have you been told that biology supports? (You could have heard things from the media, people you know, politicians, etc.)
    - *Debrief talking points*: Seeing different people use or emphasize different traits. Let's see if any one trait works across animals.
- Slide 3: Striving for consistency: "In science, the ultimate goal is to be consistent…. In the end we want all the ideas and the evidence to agree with each other. If you have one idea that doesn't fit with other knowledge or evidence, you don't just shrug it off. You try to account for it … which generally means refining the idea." (Hammer and van Zee 2006)
    - *Instructor talking points*: In science we when we come up with things like definitions of sex, we are looking for consistency across evidence. If you find evidence that doesn't fit, then we need to refine our ideas.

Case Study Activity:

- *Directions*: In pairs, Read through the case studies. Complete the provided table for your organisms. After filling out the table, answer the following question: Can any one trait tell you who can successfully reproduce with who (i.e., define the different sex types in a species)? What is your evidence for your claim?

- *Case Study 1*: White-Throated Sparrows (*Zonotrichia albicollis*)
    - White-throated sparrows have ZZ (sperm producer) and ZW (egg producer) sex chromosomes but more than sex chromosomes determine who has offspring with who. In addition to producing two types of gametes, this species has two types of physical appearances: a tan stripe over their eye and a white stripe. White-striped individuals only mate with tan-striped individuals (with only 1% of mating pairs not following this pattern). Early biologists assumed white-striped individuals produced sperm and tan-striped individuals produced eggs, but they were wrong: these stripes were found across both types of gametes. Thus, who an individual mates with is determined by gametes and appearance leading to this species being called "the bird with four sexes." If researchers only looked at gametes, they would miss many of the dynamics important for reproduction in this bird (and other species like it such as some hummingbirds & ants).
    - Stripe color is controlled by Chromosome 2 in these birds and so are other reproductive behaviors like the amount of time individuals spend caring for young or defending their territories. Scientists used to assume that these behaviors were linked to what sex chromosomes these individuals had, but in this species, they are Chromosome 2-linked behaviors. In addition, white-striped individuals have higher testosterone and estradiol (aka sex hormones) during the breeding season than tan-striped individuals, regardless of their sex chromosomes.
    - If you want to learn more check out: Maney, D. 2025. This Backyard Bird Has a Lot to Teach Us about Sex Variability. Scientific American. Link: https://www.scientificamerican.com/article/a-backyard-bird-offers-a-new-way-of-thinking-about-sexes/
- *Case Study 2*: Iberian Mole (*Talpa occidentalis*)
    - All Iberian moles have testes, tissue that can produce sperm and testosterone. In half the individuals (XX individuals) it just produces testosterone and in the other half it produces testosterone and sperm (XY individuals). In the summer through winter, all moles have the same (and very high!) levels of testosterone. Testosterone helps build muscle that these moles need to dig burrows and defend their territories.
    - In the spring, XX individuals also develop ovarian tissue (i.e., have ovotestes), tissue that can produce eggs and estrogen along with their testicular tissue. These individuals also have a clitoris that is elongated, has a foreskin, and which they urinate through, making them hard to distinguish from XY individuals based on genital morphology (this happens in elephants and hyenas too!). They mate and give birth to pups which they nurse for about a month. The ovarian tissue then reduces, and they only have testicular tissue again.
    - So, in this species it seems there are what we would typically refer to as males and also intersex individuals. At least six additional mole species also have ovotestes. Ovotestes are also common in some species of fish.
    - If you want to learn more check out: Intersexuality and the genetics of the Iberian Mole.https://knowablemagazine.org/content/article/living-world/2023/intersexuality-genetics-iberian-mole
- *Case Study 3*: Clownfish (subfamily Amphiprioninae)

    - Many species of coral reef fish are hermaphrodites. Some are simultaneous hermaphrodites, producing sperm and eggs at the same time. Others are sequential hermaphrodites, meaning they can change their sex over their lifetime. Some of these species only change sex once, and others can do it multiple times over their lifespan. Some of these species do not have sex chromosomes and others do.
    - Clownfish are well-studied examples of sequential hermaphrodites. They live in family groups on an anemone that protects them from predators. The largest individual produces eggs, the next largest produces sperm, and all the others produce neither. Their reproductive structures are called 'undifferentiated' – they have the potential to develop into sperm or egg production, but don't currently do either. These aren't babies - they can be a range of ages. They are waiting for their chance to reproduce.
    - Coral reefs are dangerous places. If the largest individual disappears, the next largest changes from producing sperm to producing eggs and the largest of the undifferentiated fish starts producing sperm. Changes start in the brain with changes in behavior (the male who was subordinate to the female now becomes aggressive) and changes in the brain trigger a cascade of hormonal changes that cause their reproductive organs to completely transform within just a few weeks. This strategy of sequential hermaphroditism means no one has to leave the safety of their anemone to find a mate.
    - This is considered an example of socially-induced sex change (i.e., fish change sex to adjust to the social dynamics in the group).
    - If you want to learn more check out: Cormier, Z. Fish are the sex-switching masters of the animal kingdom. https://www.bbcearth.com/news/fish-are-the-sex-switching-masters-of-the-animal-kingdom
- *Case Study 4*: Japanese Common Grass Yellow Butterfly (*Eurema mandarina*)
    - The common grass yellow butterfly has ZW sex chromosome system, where typically ZW individuals produce eggs and ZZ individuals produce sperm. However, on two islands in Japan, some ZZ individuals also produce eggs. Thus, sex chromosomes can't tell the whole story of sexual development in this species.
    - *Wolbachia* is a group of bacteria that is found in many insects. *Wolbachia* is passed down through generations of its hosts by being transferred inside the host's gametes (specifically eggs). *Wolbachia* often interacts with the reproductive development of its insect host. Butterflies like the Common Grass Yellow Butterfly cannot develop the internal reproductive organs needed to produce eggs without the bacteria. Butterflies who produce eggs and have ZZ chromosomes are infected with two types of *Wolbachia*, whereas butterflies who produce sperm and have ZZ chromosomes as well as butterflies who make eggs and have ZW chromosomes are infected with only one type.
    - The influence of *Wolbachia* on sexual development is felt all the way through larval development as individuals treated with antibiotics that kill *Wolbachia* at different stages develop intersex characteristics. Genetic work in this system has shown *Wolbachia* changes which segments of DNA of an important sex-determining gene are translated into proteins.
    - If you want to learn more check out: Zimmer, C. 2001. *Wolbachia*: A tale of sex and survival. https://www.science.org/doi/10.1126/science.292.5519.1093
- *Summary Table* students fill in as go through cases:

- Directions: For each species, mark (Y=yes, N=no) whether each trait below could be used to reliably determine who can produce offspring with who (i.e. define the different sex types in a species)? Reliably in this case means it works for all individuals at any time of year.

	White Throated Sparrow	Iberian Mole	Clownfish	Japanese Common Grass Yellow Butterfly
Gametes (sperm, egg)				
Sex Chromosomes (Z/W, X/Y)				
Sex Hormones				
Internal Reproductive Organs				
External Genitalia				
How many sexes would be present in this species?				

- Whole class debrief:
    - Can any of one of these traits alone tell you who can successfully reproduce with who (i.e., define the different sex types in the species) across all these species?
    - Do any traits suggest sex is binary across all these organisms?
    - If you cannot base sex classification on one trait, what might we do instead?

Wrap-up

- Sex is complicated to characterize.
- Science does not currently have a settled definition of sex that works for all animals, much less all sexually reproducing organisms because there is so much variation.
    - Illustrate this with the title and definitions of sex from three different scientific articles:
        - Smiley, K. O., Munley, K. M., Aghi, K., Lipshutz, S. E., Patton, T. M., Pradhan, D. S., & Solomon-Lane, T. K. (2024). Sex diversity in the 21st century: Concepts, frameworks, and approaches for the future of neuroendocrinology. Hormones and behavior, 157, 105445. https://doi.org/10.1016/j.yhbeh.2023.105445
        - McLaughlin, J. F., Brock, K. M., Gates, I., Pethkar, A., Piattoni, M., Rossi, A., & Lipshutz, S. E. (2023). Multivariate models of animal sex: Breaking binaries leads to a better understanding of ecology and evolution. Integrative and comparative biology, 63(4), 891-906. https://doi.org/10.1093/icb/icad027
        - Goymann, W., Brumm, H., & Kappeler, P. M. (2023). Biological sex is binary, even though there is a rainbow of sex roles: Denying biological sex is

anthropocentric and promotes species chauvinism. Bioessays, 45(2), 2200173. https://doi.org/10.1002/bies.202200173

        -

    - Some researchers are moving towards using groups of traits to define sex. These groupings do not lead to a binary understanding of sex. Other scientists focus on one trait that works a lot of the time.
- Trying to understand sex is an active area of research.

C. Annotated Bibliography: Interdisciplinary (neuro)science & technology studies

Creator: Megan G. Massa (mmassa@emory.edu)

The following is a selection of papers that I have assigned in my interdisciplinary (neuro)science and technology studies course series over the years. As such, these resources include those that are more scientific and beyond. I have broken them down into general categories that *ICB* readers may find most useful.

Historical manifestations of the sex binary (in historical order)

Laqueur T. (1990) *Making Sex: Body and Gender From the Greeks to Freud*. Harvard University Press.

- Canonical book delineating the "one-sex" and "two-sex" models in history. Most assigned portions include "Of Language and the Flesh" (pp 4-8 & 10-11), "Believing is Seeing" (pp 79-end of 82), and "Discovery of the Sexes" (pp 149-150, 151-153, and 171-174).

Stepan NL. (1993) "Race and gender: The role of analogy in science" from *The "Racial" Economy of Science*, ed. Sandra Harding. Indiana University Press.

- Chapter exploring the role of language in linking race and gender in the scientific process

Markowitz S. (2017) "Pelvic politics: Sexual dimorphism and racial difference" (reprinted and abridged from 2001) in *Queer Feminist Science Studies: A Reader*. UW Press.
https://www.jstor.org/stable/3175447

- Article demonstrating the use of sex differences in colonization and empire.

Gordon-Chimpembere, N. (2015) "Sarah Baartman, a South African Slave: A discussion on Gender and Representation." Recorded Brown Bag Lecture from Universidad Para La Paz, Costa Rica. March 9.
https://youtu.be/UgT1ctCuyyM?si=sq1_MPmUk6H2w1HP

- YouTube video/lecture briefly covering the history of Sarah Baartman (also derogatorily known as the "Hottentot Venus"). This accompanies conversations with Stepan's and Markowitz's pieces as the human impacts of these scientific frameworks.

Rubin DA. (2017) "'An unnamed blank that craved a name': A genealogy of intersex as gender" (reprinted and edited from 2012) in *Queer Feminist Science Studies: A Reader*. UW Press.
https://doi.org/10.1086/664471

- Article linking the modern day conceptualization of gender and gender role to John Money's "research" and "treatment" of intersex infants. I often pair this with videos of David Reimer's case and experience, providing ample content warnings where appropriate.

Effects of the sex binary on marginalized populations

Aghi K. et al. (2022) "Centering the needs of transgender, non-binary, and gender-diverse populations in neuroendocrine models of gender-affirming hormone therapy." *Biol Psych: Cog Neurosci and Neuroimaging* 7: 1268-1279. https://doi.org/10.1016/j.bpsc.2022.07.002Get rights and content

- Call to research medical treatments commonly used in trans populations

Davis G et al. (2016) "Giving sex: Deconstructing intersex and trans medicalization practices." *Gender and Society* 30(3): 490-514. doi: 10.1177/0891243215602102.

- Analysis of medical practices used with trans and intersex patients. First author is an intersex academic. Also paired with Clune-Taylor, C. (2019), Securing Cisgendered Futures: Intersex Management under the "Disorders of Sex Development" Treatment Model. Hypatia, 34: 690-712. https://doi.org/10.1111/hypa.12494

Malatino, H. (2017) "Gone, missing: Queering and racializing absence in trans & intersex archives" in *Queer Feminist Science Studies: A Reader*. UW Press. pp 157-171. https://doi.org/10.1515/9780295742595-015

- Provides insight into who gets included in archival space and who does not. I use this source to prompt discussion on how what we choose to study in medical and scientific research impacts what we know and who we know about.

Kessler S. (1990) "The medical construction of gender: Case management of intersexed infants." *Signs* 16(1), pp 3-26. https://doi.org/10.1086/494643

- Article interviewing medical professionals treating intersex patients.

Pagonis P. (2023) *No Body Needs to Know: A Memoir*. TOPPLE Books/Little A.

- Autobiographical book from an intersex activist

Semenya C. (2023) "Running in a Body That's My Own." *New York Times*, Oct 21. https://www.nytimes.com/2023/10/21/opinion/running-body-semenya.html?mwgrp=a-dbar&unlocked_article_code=1.KU0.kpsO.uL-r6jNTlSMQ&smid=url-share

- Excerpt from Olympic runner Caster Semenya's book on her experience with gender testing in the IOC and the medical labels placed upon her

Weigel AR. (2023) *Inverse Cowgirl: A Memoir*. HarperCollins.

- Autobiographical book from an intersex activist

Gender impacts on scientific schemas

Ahnesjö I et al. (2020) "Considering gender-biased assumptions in evolutionary biology." *Evolutionary Biology* 41(1): 1-5. https://doi.org/10.1007/s11692-020-09492-z

- Article arguing that gender is present in science and providing guidelines for future practice.

Fausto-Sterling A. (2014) “The bare bones of sex: Part 1 – Sex and gender” in *Women, Science, and Technology: A Reader in Feminist Science Studies*. 3rd edition. Routledge. pp 272-296

- Article demonstrating entanglement of gender and sex via the impact of gender on biology (bones) and advocating for a more dynamic systems theory approach.

Hoffman GA & Blum R. (2016) “Neurosexism and neurofeminism” *Compass* 11(11):716-729. https://doi.org/10.1111/phc3.12357

- Overview sexist bias in neuroimaging studies at the levels of data collection, analysis, presentation, and interpretation, with particular emphasis on “hard-wiring.”

Martin E. (1991) “The egg and the sperm: How science has constructed a romance based on stereotypical male-female roles.” Signs 16(3), pp 485-501. https://doi.org/10.1093/oso/9780198751458.003.0008

- Article examining word choice, etc., across biology text books to reveal gendered assumptions in the way science portrays fertilization. This is an excellent resource to teach students the literary tool of “close reading” via sentence structure, syntax, and word choice.

Milam E. (2014) “Making males aggressive and females coy: Gender across the animal-human boundary” in Women, Science, and Technology: A Reader in Feminist Science Studies. 3rd edition. Routledge. 2014. pp 206-222. https://doi.org/10.1086/664474

- Article demonstrating the gendered assumptions inherent in sexual selection theory and animal behavior.

Richardson SS. (2017) “Sexing the X: How the X became the ‘female chromosome’” (reprinted and abridged from 2012) in *Queer Feminist Science Studies: A Reader*. UW Press. pp 30-42. https://doi.org/10.1515/9780295742595-005

- Article demonstrating the gendered assumptions in the science and stories about the X chromosome

Shansky R. (2019) “Are hormones a ‘female problem’ for animal research?” *Science* 364(6443):825-826. https://doi.org/10.1126/science.aaw7570

- Commentary on the gendered conceptions that lead to bias in scientific questions and experimental design concerning females.

van den Wijngaard M. (1997) *Reinventing the Sexes: The Biomedical Construction of Femininity and Masculinity*. Indiana University Press.

- Book exploring the construction of gendered understandings through scientific study of sex differences and the organizational/activational hypothesis

Data undermining gendered assumptions

Becker JB, Prendergast BJ, & Liang JW. (2016) “Female rats are not more variable than male rats: a meta-analysis of neuroscience studies.” *Biology of Sex Differences*. 7(1):34. https://doi.org/10.1186/s13293-016-0087-5

- Scientific article demonstrating no difference in variability between female and male rats

Prendergast BJ, Onishi KG, & Zucker I. (2014) “Female mice liberated for inclusion in neuroscience and biomedical research.” *Neurosci & Behav Rev*. 40: pp 1-5. https://doi.org/10.1016/j.neubiorev.2014.01.001

- Scientific article demonstrating no difference in variability among female and male mice

Nugent BM et al. (2015) “Brain feminization requires active repression of masculinization via DNA methylation.” *Nat Neurosci*. 18: 690-687. https://doi.org/10.1038/nn.3988

- Article demonstrating active epigenetic maintenance of testosterone-induced organizational effects in the brain

Rocks D, Cham H, & Kundakovic M. (2022) “Why the estrous cycle matters for neuroscience.” *Biology of Sex Differences* 13(62): pp1-14. https://doi.org/10.1186/s13293-022-00466-8

- Scientific article demonstrating importance of estrous cycle variation while also confirming no general difference in female-male variability overall. Any evidence of female-male variability does not predict estrous cycle importance to the phenotype being investigated.

Smarr B & Kriegsfeld LJ. (2022) “Female mice exhibit less overall variance, with a higher proportion of structured variance, than males at multiple timescales of continuous body temperature and locomotive activity records.” *Biology of Sex Differences* 13(41): 2-10. https://doi.org/10.1186/s13293-022-00451-1

- Scientific article demonstrating no difference in female and male variability overall but more predictable variability for females day-to-day.

Zhao F et al. (2017) “Elimination of the male reproductive tract in the female embryo is prompted by COUP-TFII in mice.” *Science* 357(6352): 717-720. https://doi.org/10.1126/science.aai9136

- Scientific discovery of COUP-TFII. Given elimination of this gene results in Wolffian duct persistence, this paper destabilizes the idea of female as default (and thus female passivity).

The social construction of sex

Fausto-Sterling A. (2000) *Sexing the Body*. Basic Books.

- Book exploring the social construction of sex, gender, and sexuality. Most used excerpt is Chapter 1: Dueling Dualisms.

Oudshoorn N. (1994) “Sex and the body” from *Beyond the Natural Body: an archeology of sex hormones*. Routledge.

- Book detailing the social history of sex hormones. Most frequently-assigned excerpt is “Sex and the Body” (pp 6-9).

Oyěwùmí O. (1997) *The Invention of Women: Making an African Sense of Western Gender Discourses*. University of Minnesota Press.

- Book critiquing on Western perspectives of gender and the impact of colonization. Most assigned excerpts are “Social Orders and Biology: Natural or Constructed?” (pp 8-11) and “The ‘Sisterarchy’: Feminism and Its ‘Other’” (pp.11-17).

Questioning the sex binary

Clune-Taylor C (2021) “Is sex socially constructed?” *The Routledge Handbook of Feminist Philosophy of Science*. Routledge. pp 187-200

- Argument that the scientific category of sex is not a biological given but is constructed and reconstructed through discourse and scientific practice

Massa MG, Aghi K, and Hill MJ (2023) “Deconstructing sex: Strategies for undoing binary thinking in neuroendocrinology and behavior.” *Hormones and Behavior* 156(105441): 1-7. https://doi.org/10.1016/j.yhbeh.2023.105441

- Argument that the sex binary heuristic is incoherent and gendered. Provides questions and paths forward for scientific researchers to consider.

McLaughlin JF et al. (2023) “Multivariate Models of Animal Sex: Breaking Binaries Leads to a Better Understanding of Ecology and Evolution.” *Integrative & Comparative Biology* 63(4):891-906. https://doi.org/10.1093/icb/icad027

- Argument for multivariate models of sex

Pape M, Miyagi M, Ritz SA, Boulicault M, Richardson SS, & Maney DL (2024) “Sex contextualism in laboratory research: Enhancing rigor and precision in the study of sex-related variables.” *Cell* 187(6): 1316-1326. https://doi.org/10.1016/j.cell.2024.02.008

- Description of approaches to research on sex-associated variables that push beyond binary and gendered thinking

Rehmann-Sutter C et al. (2023) “Is sex still binary?” *medizinische genetic* 35(3):173-180 https://doi.org/10.1515/medgen-2023-2039

- Provides an overview of the arguments for and against binary sex while also giving scientific and other interdisciplinary evidence for nonbinary manifestations and usages

Approaches to sex and gender research

Baker Z & Hayssen V. (2024) “A practical guide to avoiding biased communication in reproductive biology.” *Integrative and Comparative Biology* 64(6):1717-1733. https://doi.org/10.1093/icb/icae138

- Argues for codifying and neutralizing gendered language and approaches to figures.

Bentley VA. (2021) “Feminism and cognitive science.” *The Routledge Handbook of Feminist Philosophy of Science*. pp 328-339

- Book chapter that reviews preceding feminist critiques and approaches to cognitive science, including but not limited to: reporting similarities, specific physiologies, socialization, statistical practices, non-essentialism, new questions, multidimensional frameworks, and mosaicism.

Bentley VA (2025) “A feminist standpoint for cognitive neuroscience.” *Synthese* 206(1). https://doi.org/10.1007/s11229-025-05106-3

- Article outlining the current framework for sex differences research in cognitive science as sex essentialist before developing a feminist framework that centers situated knowledge and epistemic advantage thesis.

DeCasien AR et al. (2022) "Sex differences in the human brain: a roadmap for more careful analysis and interpretation of a biological reality." *Biology of Sex Differences* 13(1):43. https://doi.org/10.1186/s13293-022-00448-w

- Commentary on large-scale sex differences studies with suggestions for better approaches

DuBois LZ & Shattuck-Heidern H. (2021) "Challenging the binary: Gender/sex and the bio-logics of normalcy." *Am J Hum Biol.* 33:e23623. https://doi.org/10.1002/ajhb.23623

- Discussion of biological variation associated with gender/sex and the search for difference before providing research design and survey question methodologies for incorporation in human studies.

Eliot L et al. (2023) "Why and how to account for sex and gender in brain and behavioral research." *J Neurosci.* 43(37):6344-6356. https://doi.org/10.1523/jneurosci.0020-23.2023

- Guidance for statistical methods, key sex and gender covariates, interpretations, and science communications in brain and behavioral research

Holmes MM & Monks DA. (2019) "Bridging sex and gender in neuroscience by shedding a priori assumptions of causality." *Front. Neurosci.* 13:475. https://doi.org/10.3389/fnins.2019.00475

- Argue for a revaluation of definitions of sex and gender which prematurely dichotomize biological and the social in animals

Joel D & Fausto-Sterling A. (2016) "Beyond sex differences: New approaches for thinking about variation in brain structure and function." *Phil Trans R Soc B* 371:20150451. https://doi.org/10.1098/rstb.2015.0451

- Argument for studying sex effects in context and development/analysis of data that take into account brain heterogeneity

Kaiser A. (2012) "Re-conceptualizing "sex" and "gender" in the human brain." *Zeitschrift für Psychologie*. 220(2): 130-136. https://doi.org/10.1027/2151-2604/a000104

- Article advocating for the use of the term "sex/gender" due to the embodiment and entanglement of the concepts in the human brain

McCarthy MM et al. (2012) "Sex differences in the brain: The not so inconvenient truth." *J Neuro* 32(7):2241-2247. https://doi.org/10.1523/jneurosci.5372-11.2012

- Strategies to neuroendocrine research (complete with flowcharts)

McCarthy MM & Konkle ATM. (2005) "When is a sex difference not a sex difference?" *Front. In Neuroendocrinology* 26(2):85-102. https://doi.org/10.1016/j.yfrne.2005.06.001

- Disambiguation between sex differences and dimorphisms across various brain structures and behaviors

Nikoleyczik K. (2012) "Towards diffractive transdisciplinarity: Integrating gender knowledge into the practice of neuroscientific research." *Neuroethics* 5(3):231-245. https://doi.org/10.1007/s12152-011-9135-3

- Methodological considerations for cognitive neuroscience studies looking at sex and gender

Pape M et al. (2024) "Sex contextualism in laboratory research: Enhancing rigor and precision in the study of sex-related variables." *Cell* 187(6):1316-1326. https://doi.org/10.1016/j.cell.2024.02.008

- Argues for a sex contextual approach, creating *a priori* hypothesis about sex, making correct statistical comparisons, and increased precision

Rich-Edwards JW & Maney DL. (2023) "Best practices to promote rigor and reproducibility in the era of sex-inclusive research." *eLife* 12:e90623. https://doi.org/10.7554/eLife.90623

- Makes recommendations for applying the NIH's "4Cs of Studying Sex to Strengthen Science: Consideration, Collection, Characterization, and Communication" for inclusive sex research.

Richardson SS (2021) "Sex contextualism." *Philos Theor Pract Biol* 13(9). https://doi.org/10.3998/ptpbio.2096

- Delineation of a sex contextualist approach that rebuffs sex essentialism through incorporation of innumerable sexes into a research study based on timing dynamics and variable of interest.

Rippon G et al. (2014) "Recommendations for sex/gender neuroimaging research: Key principles and implications for research design, analysis, and interpretation." *Front. Human Neurosci.* 8:650. https://doi.org/10.3389/fnhum.2014.00650

- Methodological and interpretation considerations of overlap, mosaicism, contingency, and entanglement for neuroimaging research

Rippon G, Losse K, & White S. (2024) "Impression management in sex and gender neuroscience research reporting: the MAGIC guidelines." *Nat Comm.* 15(1):2826. https://doi.org/10.1038/s41467-024-47261-0

- Adapts the MAGIC framework (magnitude, accuracy, generalizability, inflation, and credibility) for sex/gender research in neuroscience

Sanchis-Segura C, Forn C, & Wilcox RR. (2026) "From significant to meaningful: ATOMizing the study of sex differences and similarities." *Frontiers in Neuroendocrinology* 80:101228. https://doi.org/10.1016/j.yfrne.2025.101228

- Describes an alternative statistical method, the Threshold Probability of Superiority, to means testing as an application of ATOM principles (Accept uncertainty, be Thoughtful, be Open, be Modest).
- Commentary on problems with current statistical testing found: Sanchis-Segura C & Wilcox RR. (2024) "From means to meaning in the study of sex/gender differences and similarities." *Frontiers in Neuroendocrinology* 73:101133. doi: 10.1016/j.yfrne.2024.101133

Smiley KO et al. (2024) "Sex diversity in the 21st century: Concepts, frameworks, and approaches for the future of neuroendocrinology." *Hormones and Behavior* 157(105445): 1-23. https://doi.org/10.1016/j.yhbeh.2023.105445

- Provides new frameworks for a multivariate sex analysis

Springer KW, Stellman JM, & Jordan-Young RM. (2012) "Beyond a catalogue of differences: A theoretical frame and good practice guidelines for researching sex/gender in human health." *Social Sci & Med* 74(11):1817-1824. https://doi.org/10.1016/j.socscimed.2011.05.033

- Elimination of sex itself as a biological mechanism and experimental considerations for entanglement of gender and sex

Subramaniapillai S et al. (2024) "Sex and gender in health research: Intersectionality matters." *Frontiers in Neuroendocrinology* 72:101104. https://doi.org/10.1016/j.yfrne.2023.101104

- Argues for an intersectional approach that marries biological, environmental, and social contributors to sex-linked disparities in health

van Anders SM. (2015) "Beyond sexual orientation: Integrating gender/sex and diverse sexualities via sexual configurations theory." *Arch Sex Behav* 44:1177-1213. https://doi.org/10.1007/s10508-015-0490-8

- New approach to collecting information on sex, gender, gender/sex, and sexual orientation from human participants

van Anders SM et al. (2025) "Feminist and queer science: Principles for research with gender, sex, and sexuality in psychology and beyond." *Psychology of Sexual Orientation and Gender Diversity* 12(2):181-200. https://doi.org/10.1037/sgd0000646

- Provides a primer to the application of feminist and queer studies to science research.

Waters AS. (2001) "Language matters - a metaphysic of nondiscreet nonbinary dualism." *American Philosophical Newsletter on American Indians in Philosophy*. pp 5-10.

- Chapter exploring Indigneous frameworks of gender that do not inscribe hierarchy

Watkins A & DiMarco M (2025). "Sex eliminativism." *Biology & Philosophy* 40(1):2. https://doi.org/10.1007/s10539-024-09972-y

- Argues that the category of sex should be wholly eliminated from scientific research practice.

Williams JS et al. (2023) "Considering hormones as sex- and gender-related factors in biomedical research: Challenging false dichotomies and embracing complexity" *Hormones and Behavior* 156:105442. https://doi.org/10.1016/j.yhbeh.2023.105442

- Reviews gendered thinking in biomedical science and dynamic reality of hormones before arguing for a sex contextualist approach and mindful data interpretation.

D. Activity: Teaching Diversity Through Intersex

Creator: Megan G. Massa (mmassa@emory.edu)

Timing: 45 minutes to 1 hour

Context Developed for:

- Mid-level course
- Discussion-based or lecture-based course
- Flexible assessment course

Learning Goals for Activity

- State the process of sex determination and sexual differentiation
- Infer type of variability in sex physiology based on developmental mechanism
- Determine phenotypic output from description of intersex trait
- Recognize the inherent variability of sexed phenotypes

Activity Components:

Slides, activity, and assignment rubrics available in online repository:
https://www.betterbiologynetwork.org/

Lecture (slides available in repository)

- Slides 1-4: Sex determination is the "selection" of the gonad from an undifferentiated state. Ovary and testis development are both active genetic processes that mutually inhibit one another. *SRY* on the Y chromosome turns on temporally before either *FOXL2* (in the ovarian pathway) or *SOX9* (in the testicular pathway).
- Slide 5-6: Sexual differentiation is the development of a sexed body plan.
- Slides 7-8: Development of the testis from a primordial gonad results in the release of anti-Müllerian hormone (AMH) and testosterone (T). AMH works to degrade the Müllerian ducts while T works to promote the maturation of the Wolffian system. T also, through its conversion to dihydrotestosterone (DHT), promotes the development of penis and scrotum from the undifferentiated genital mound.
- Slide 9: Development of the ovary from a primordial gonad does not (to our knowledge) release any hormones during this developmental window. Lack of AMH allows Müllerian ducts to persist, and ovarian presence is somehow related to the expression of *COUP-TFII* in the undeveloped Wolffian system, degrading it. Lack of T results in the development of clitoris and labia from the undifferentiated genital mound.
- Slide 10: Flow chart depicting combined processes of sex determination and sexual differentiation.

- Slide 11: Is sex a spectrum? Depends on what sex you're talking about. Sex phenotypes derived from independent parts such as chromosomes or internal genitalia have discrete configurations of outputs. On the other hand, homologous structures created from the same primordial tissue tend to exhibit a bimodal spectrum of variability. Phenotypes downstream of a plethora of biological etiologies exhibit true spectral variation given their multifactorial nature.
- Slide 12: (Extra information required for next activity) Gonadal hormones are all derived from cholesterol. The hormones that a tissue produces is dependent on what enzymes are expressed. If a tissue normally produces a hormone type but the enzyme responsible for that conversion is not present or not working, the tissue may "shunt" hormonal production down another pathway and produce another class of steroid hormone given enzyme expression.

Intersex Chart Activity

- *Directions*: Working with a partner or alone, predict the phenotypic output for the different levels of sex that would be observed given the biology to the left of each row. Examples of possible configurations are provided below each heading. Include a brief reasoning beneath each answer. Submit on Canvas. You will be able to redo this worksheet until you have correctly answered all boxes.

Lecture Wrap-Up

- Slides 13-15: We have gone through the "old view," which primarily located sex as a linear pattern from chromosomes to hormones to phenotypes. This view still works for some outputs, as hormonal impacts on the brain include alterations in neurogenesis, apoptosis, molecular phenotype, cytoarchitecture, and circuitry.
- Slides 16-19: However, this is not the whole story. The "new view" has shown that chromosomes themselves can alter phenotypes, as in the case of gynandromorphic animals, as well as sex-influenced life experiences.

Assessment Options

- **Intersex Trait "InfoPoster"**: Research and produce a poster or infographic on the biological reasons behind and the firsthand personal experience of living with a specific intersex trait. While this project is very much concerned with biology, the focus is on recentering intersex people as makers of their own narratives. You can select any modality you wish. Regardless of modality, your project must contain accurate biological information and (a) first-hand account(s) from intersex individuals throughout.
- **Intersex Educational Tool**: Develop an educational tool for children with intersex variations: what they are (generally) and how some common ones come about. You may choose any authoritative position (e.g., an Emory undergrad, a doctor, an activist) and any audience (e.g., parents of intersex children, the intersex child themselves, an elementary school classroom, your family). Ultimately, you are responsible for creating a tool that is a) factual and b) considers all that we have begun to talk about in the last two modules, including the interplay between sex and gender along with the idea that our schemas may be socially-based and employ "bio-logics." You

can select any modality for your project. Since it is a tool, it may require that you submit it with instructions on how to use said tool. Because this is an informational and educational tool, you should include references for your *audience* to do more research if they want to learn more. These may not necessarily be where you got your information from. References of where information was garnered should be included in-text and a reference list should be included in the tool in whatever way makes sense for the modality and should be aimed at the *presenter* of the tool.

E. Syllabus: “Diversity of Sex” Course

Creator: Karen M. Warkentin (kwarken@bu.edu)
500-Level, 4 credits

Course Description

The evolution of sex was a major event in the history of life, facilitating adaptation and the diversification of life, including the evolution and differentiation of sexes and a wide variety of sex-related phenotypes. The biology of sex thus offers opportunities to study fascinating, cutting-edge questions in evolutionary and developmental biology. However, our understanding of the biology of sex and sexes has been shaped by human social expectations and biases, changing dramatically with social changes and the incorporation of different perspectives and research questions; this expansion of perspectives is ongoing. The biology of sex thus also serves as an excellent area to critically examine science as a human process.

In this course, we will attempt to build a more inclusive understanding of the diversity of sexual biology by examining foundational questions about sex as well as new research that challenges conventional perspectives and is shifting our understanding of sexual variation, in humans and other organisms. One goal is to improve our ability to ask questions and design and evaluate research, considering all the variation. A second goal is to develop and practice inclusive ways to study and discuss biology, respecting human variation. A third goal is to develop and practice communication skills to share and consider this information with others.

Students will actively participate in finding and sharing relevant papers and determining topic coverage through the semester. We will share and refine our growing knowledge and understanding through multiple class presentations and extensive discussion of materials.

Course objectives

1. Build core knowledge of sex-related evolutionary and developmental biology, including

- current understandings
- areas and concepts currently in flux or in debate
- historical changes in sex-related research and their relation to other social changes

2. Practice and build skills to

- integrate knowledge across disciplinary areas and approaches
    - improve understanding, identify gaps and discrepancies
    - develop creative approaches to answer questions
- communicate science and communicate about science
    - clearly present research results to other scientists (oral/signed)
    - effectively engage in discussions of research itself and in relation to social context
- critically examine science as a human process
    - ask questions, design and evaluate research in ways that include all the variation

    - consider ways to acknowledge & minimize impacts of bias, while building knowledge
    - include diverse scientists and human perspectives in science
- use oral and/or signed communication to responsibly and effectively present and discuss scientific material about the diversity of sexual biology.
- attend and respond thoughtfully to others in interactive oral/signed communication on sex-related topics that may be scientifically contested and/or relevant to personal identities.
    - speak/sign effectively in situations ranging from structured presentations to class discussions.

Instructor's approach to this material

I am broadly interested in developmental and evolutionary diversity and seek to understand the causes and consequences of patterns of phenotypic variation. The amazing diversity of reproductive and sexual biology—of which binary differences are just one part—has been created by fundamental, general mechanisms of developmental and evolutionary biology, thus offering excellent examples of these processes. I consider that both oversimplification of more complex variation and overemphasis on sex as an explanatory factor sometimes misleads us. However, the current focus on "sex differences" in biology and medicine is part of a necessary attempt to rectify a historical focus on males, which has led to a disproportionate lack of information on females. An alternative approach to this problem is sometimes to simply focus on females. Moreover, the biology of sex is often presented in (over)simplified ways that reflect and reify social norms and limit opportunities to understand biological diversity. Human social norms also affect the questions biologists ask – and don't ask – and how research results are interpreted. Thus they affect the current state of our knowledge. The more I learn about sexual and reproductive biology, the more wary I become of (over)generalizations. Some things I was taught – and that I myself taught to prior students – have turned out to be either wrong or simply not general, often in ways that centered (a subset of) mammalian biology as the norm, or that fit societal notions better than do more complex natural patterns. Sometimes my understanding has been altered by new research and publications. Other times the information was already in the literature, but not widely known. I am sure there are things I now think I know that will also turn out to be wrong. In this class, we will examine our changing understanding of sexual and reproductive biology and attempt to develop approaches for working with this material – both in teaching and in research – that are less susceptible to human biases.

Course structure

Two class meetings per week, 1h 45m each. Most classes will be a mixture of two or more activities, including lectures, discussions, and student presentations with peer feedback.

Class topics and assigned readings

1. *Introduction, class planning, backgrounds & priorities*

I. Foundations – developmental & evolutionary approaches to variation

2. *Human variation and the complexity of sexual development*

- Ainsworth C. 2015. Sex redefined: the idea of two sexes is simplistic. Nature 518:288–291.
- Fausto-Sterling A. 2015. Concept of multiple sexes is not new. Nature 519:291
- Montañez A. 2017. Beyond XX and XY. Sci Amer 317:50–51.

3. *Gender/sex, challenges to the gender binary, and the importance of language*

- Hyde JS, Bigler RS, Joel D, Tate CC, van Anders SM. 2019. The future of sex and gender in psychology: Five challenges to the gender binary. Amer Psychol. 74:171-193. https://doi.org/10.1037/amp0000307
- van Anders SM. 2014. Nomenclature and knowledge-culture, or, we don't call semen 'penile mucous.' Psychology & Sexuality. 5:349–356. https://doi.org/10.1080/19419899.2013.835743
- Hayssen V, Orr T. 2017. Reproduction in Mammals: The Female Perspective. Chapter 1. Baltimore: Johns Hopkins University Press.

4. *Phenotypic plasticity and mechanisms of development and evolution*

- Sultan SE. 2021. Phenotypic plasticity as an intrinsic property of organisms In Pfennig DW (ed) Phenotypic Plasticity and Evolution: Causes, Consequences, Controversies. CRC Press p. 3–24.

5. *Cross-sexual transfer in development and evolution*

- West-Eberhard MJ. 2003. Developmental Plasticity and Evolution Chapter 15. Cross-sexual transfer. Oxford University Press
- Anderson AP, Falk JJ. 2023. Cross-sexual transfer revisited. Integr Comp Biol 63:936–945. https://doi.org/10.1093/icb/icad021

II. Origins, prevalence, and loss of sex/es

6. Evolution of (meiotic) sex

- Brandeis M. 2021. Were eukaryotes made by sex? Sex might have been vital for merging endosymbiont and host genomes giving rise to eukaryotes. Bioessays 43:e2000256. https://doi.org/10.1002/bies.202000256

7. Maintenance, loss, and plastic vs. obligate use of sex

- Burke NW, Bonduriansky R. 2017. Sexual conflict, facultative asexuality, and the true paradox of sex. Trends Ecol Evol 32:646-652. https://doi.org/10.1016/j.tree.2017.06.002
- Lehtonen J, Jennions MD, Kokko H. 2012. The many costs of sex. Trends Ecol Evol 27:172–178. https://doi.org/10.1016/j.tree.2011.09.016

8. Origins of sexes, losses of sexes: Anisogamy

- Gorelick R, Carpinone J, Derraugh LJ. 2017. No universal differences between female and male eukaryotes: Anisogamy and asymmetrical female meiosis. Biol J Linn Soc 120:1–21. https://doi.org/10.1111/bij.12874

III. Regulating sex expression – Sex determination & organization in bodies

9. Evolution and diversity of sex-determination mechanisms

- Bachtrog D, Mank JE, Peichel CL, Kirkpatrick M, Otto SP, et al. 2014. Sex determination: Why so many ways of doing it? PLoS Biology 12:e1001899. https://doi.org/10.1371/journal.pbio.1001899
- Capel B. 2017. Vertebrate sex determination: Evolutionary plasticity of a fundamental switch. Nat Rev Genetics 18:675–689. https://doi.org/10.1038/nrg.2017.60

10–11. Sexual system diversity: Organizing sexes in bodies

- Leonard JL. 2018. Transitions Between Sexual Systems: Understanding the Mechanisms of, and Pathways Between, Dioecy, Hermaphrodism and Other Sexual Systems, Chapter 1. The evolution of sexual systems in animals. Springer.

IV. Sex diversification – Patterns and mechanisms

12. Limitations of classic theory on sex role divergence

- Tang-Martinez Z. 2016. Rethinking Bateman's principles: challenging persistent myths of sexually reluctant females and promiscuous males. J Sex Res 53:532-559. https://doi.org/10.1080/00224499.2016.1150938

13. Natural, social, and sexual selection on females and males

- Tobias JA, Mongomerie R, Lyon BE. 2012. The evolution of female ornaments and weaponry: Social selection, sexual selection and ecological competition. Phil Trans R Soc B 367: 2274-2293. https://doi.org/10.1098/rstb.2011.0280

14. Mate choice as a process: Mechanisms, contextuality, mutuality

- Rosenthal GG. 2017. Mate Choice: The Evolution of Sexual Decision Making from Microbes to Humans Chapter 1. Mate choice and mating preferences: an overview. Princeton.

15. Sex and polymorphism

- Mank JE. 2023. Sex-specific morphs: the genetics and evolution of intra-sexual variation. Nat Rev Genet 24:44-52. https://doi.org/10.1038/s41576-022-00524-2
- Brooks, R. 2019. Queer birds: Avian sex reversal and the origins of modern sexology. Viewpoint 119:10–11

16. Evolution of cooperative breeding and altered selection on sexes

- Burkart JM, van Schaik C, Griesser M. 2017. Looking for unity in diversity: Human cooperative childcare in comparative perspective. Proc R Soc B. 284 (1869): 20171184. https://doi.org/10.1098/rspb.2017.1184

- Ben Mocha Y, de Gialluly SS, Griesser M, Markman S. 2023. What is cooperative breeding in mammals and birds? Removing definitional barriers for comparative research. Biol Rev 98:1845–1861. https://doi.org/10.1111/brv.12986

17. Humans as cooperative breeders: Implications

- Hrdy, SB, 2009. Mothers and Others: The Evolutionary Origins of Mutual Understanding Chapter. 3 Why it takes a village. Belknap.

18. A plant-centered perspective on sexual diversity

- Moore JC, Pannell JR. 2011. Sexual selection in plants. Curr Biol 21:R176–182. https://doi.org/10.1016/j.cub.2010.12.035
- Haufler CH, Pryer KM, Schuettpelz E, Sessa EB, Farrar DR, Moran R, Schneller JJ, Watkins JEJ, Windham MD. 2016. Sex and the single gametophyte: Revising the homosporous vascular plant life cycle in light of contemporary research. Bioscience 66:928–937. https://doi.org/10.1093/biosci/biw108

19. Work day: *Peer feedback on project drafts*

V. Diversity in sexual behavior

20. Non-reproductive sexual behavior in animals

- Wolff JO, Macdonald DW. 2004. Promiscuous females protect their offspring. Trends Ecol Evol 19:127-134. https://doi.org/10.1016/j.tree.2003.12.009
- Lukas D, Huchard E. 2014. The evolution of infanticide by males in mammalian societies. Science 346:841-844. https://doi.org/10.1126/science.1257226

21. Same-sex sexual behavior in animals

- Monk JD, Giglio E, Kamath A, Lambert MR, McDonough CE. 2019. An alternative hypothesis for the evolution of same-sex sexual behavior in animals. Nature Ecol Evol 3:1622-1631
- Bailey NW, Zuk M. 2009. Same-sex sexual behavior and evolution. Trends Ecol Evol 24:439-446. https://doi.org/10.1038/s41559-019-1019-7
- Vasey PL, Sommer V. 2006. Homosexual behavior in animals: topics, hypotheses and research trajectories. in Sommer & Vasey Homosexual Behavior in Animals: An Evolutionary Perspective Cambridge

VI. Integration

21. Conceptual integration: Presentation & discussion of Warkentin & al. work-in-progress

- Warkentin KM, Falk JJ, Casper AMA. 2026. Evolution, development, and the incoherence of sex: A framework of multiple sex concepts. Integr Comp Biol 66:icag133. https://doi.org/10.1093/icb/icag113

22. Work day: *Sex Diversity Portfolio Teamwork*

VII. Sex diversity scholars' symposium

23–26. Student final project talks (4 classes)

VIII. Finale

27. Sharing & discussion of Sex Diversity Portfolios; course wrap-up & evaluation

Assignments and Assessment

Effort, practice and feedback is necessary for learning, but the role and value of grades *per se* in learning is debatable. We'll discuss feedback and grading in our first class and make some collective decisions about the use, nature, and amount of self-assessment, peer assessment, and professorial assessment, starting with the structure below.

1. **Class participation** (20%). Participation in the class learning community through pre-lecture comments on readings through the online social annotation platform *Perusall*, in-person discussion of readings and presented material, and peer feedback on classmates' work. **Grading** will be substantially based on your mid-semester and final reflection & self-assessment, but may be adjusted in cases of discrepancy with submitted materials and classroom contributions.

- **Benefits** – Strengthen your communication skills, hone your understanding of materials, build community, mutually aid your classmates and receive aid from them.

2. **Three short talks** (30%). **i)** Choose a candidate paper for each presentation with input from the professor., **ii)** prepare and present information from it in a 5-minute talk, answer questions/engage in discussion about it, **iii)** submit a reflection on your talk (via google form), receive peer and professor feedback. **Grading** based on a combination of self-assessment, peer feedback, and professorial evaluation. First talk: completion grading (i.e., do the 3 steps = full credit). Talks 2 & 3: Graded based on a combination of self-assessment, peer feedback, and holistic professorial evaluation.

- **Benefits for presenter** – Explore an area of literature, practice finding and evaluating research papers, improve oral & visual presentation skills, build skills in distilling a body of research into a concise format, share something interesting & useful with others.
- **Benefits for others** – Exposure to more, and more diverse research than you find and read yourself; practice evaluating and giving feedback on presentations helps build your own presentation skills.

3. **Project and final presentation** (30%). Research and longer-form presentation on a topic of interest, within the scope of the course: i) discuss your interests with instructor, ii) submit a short topic application with initial bibliography, iii) research the topic and prepare a draft presentation for review & feedback, iv) provide peer reviews to your colleagues, v) revise and improve your presentation and present it to the class at the end of the semester. Your project structure and format is flexible, pending discussion with professor to ensure it is suited to the topic and available literature – students may choose to prepare reviews, research proposals, critiques, etc. Talk with me about your ideas! I encourage you to communicate with me and with each other about your projects at any stage of the process – the benefits of

collegial input are not restricted to formal peer review. *Note: talk length determined with students, based on class size and the number of sessions available. We will have 3 talks per 105 minute class – so a maximum of 35 min for presentation, Q&A, and reviewer note-taking. Talks should be about 20 minutes, and not more than 25.* Grading based on a combination of self-assessment, peer feedback, and holistic professorial evaluation, considering all stages.

- **Benefits for researchers/presenters** – Explore an area of interest in greater depth than is possible for the whole class to do, practice integrating material from multiple sources into a coherent presentation, receive feedback at multiple stages to improve your understanding of your topic of interest and your communication about it.
- **Benefits for others** – Learn about areas that others researched by performing peer reviews and attending and discussing their final presentations; improve your own presentation skills by evaluating others; practice engaging collegially in the scientific community.

4. **Sex Diversity Toolkit – Group portfolio and individual reflection** (20%). What were the most useful things you learned in this class and what materials were most valuable for your personal knowledge growth? Working in small groups, you will prepare a "toolkit" portfolio with 5 contributions from each member and a short collective introduction and summary. Working individually, you will write a reflection statement explaining why you chose each of the concepts and materials you did, and what their value is for you going forward. **Graded** by professor, informed by your final reflection and self-assessment.

- **Benefits** – This exercise will help you reflect on and consolidate your knowledge from the class and provide an opportunity to engage with your peers in discussions of your different and/or shared selections. It will also help inform my choices in future iterations of this class.

Community of Learning Course Policies

Mutually respectful learning environment This course may challenge participants' assumptions about sex, gender, and sexuality, as well as our prior understanding of the biology of these topics, particularly for areas that are rapidly changing. We will discuss material that may have personal and/or social/political dimensions that different members of the class experience or relate to differently. **Diversity of perspective and opinion is expected and welcome in the class.** Social science research supports that such diversity improves information processing, complex thinking, problem-solving, creativity, etc., and that interacting respectfully with others unlike ourselves improves the performance of both majority group members and minorities; thus human diversity improves science and other fields. To enable us all to reap the benefits of our intellectual, experiential, and identity diversity, we will commit to mutually respectful discourse.

Preparation, personal responsibility, and workload. To get the most out of course meetings, students must (1) ***come prepared***: read the assigned papers thoughtfully, comment in Perusall, and bring your ideas, insights, questions and critiques to discuss with colleagues in class; (2) ***take responsibility for your own learning***: communicate your interests and priorities to help shape our topic coverage and your insights and confusions as we discuss those topics, dig deeply into the research you will communicate to others; (3) ***work hard***: we will all invest substantial effort into finding and evaluating literature, reading,

working to understand and integrate materials, and improving our ability to communicate about complex and challenging topics. I expect that for most class meetings we will all read at least one paper, sometimes more (depending on length). Everyone will do additional reading to find papers to present, multiple times through the semester. **This is a 500-level class, designed for graduate students and highly motivated seniors, so expectations are high, but *please tell me* if you find the reading unmanageable.**

F. Teaching Sex-Diversity through Writing Activities

Creator: Jay J. Falk (j.jinsing@gmail.com)

Annotated Bibliography Blogpost Assignment

You will write 2 annotated bibliography blogposts. Each post should be 600-700 words.

Learning Objectives:

- Understand how the process of writing can be used as a tool to learn
- Begin the practice of reading and finding scientific research papers
- Learn to read actively with thought rather than passively absorbing

We often think of writing as a way to place pre-existing thoughts onto paper. However, the process of writing is also critical for organizing our thoughts and solidifying them into more coherent ideas. For the duration of this course, you will become experts on a type of reproductive system in an animal. This reproductive system will likely be unfamiliar to you. So how can you start this endeavor? Reading is of course one place to start, but by simultaneously writing you can enhance our understanding even further.

I have selected research papers on each of your topics. Your first post will be on the paper I provided, but you need to find the second paper on your own. Read the paper while considering the following questions:

- What was the purpose of doing the study? What question are the scientists trying to answer?
- What did the scientists do to answer this question?
- What did they find?
- What do the authors conclude based on their findings?

An annotated bibliography is a summary and reflection on a journal article. Your assignment will be to write an annotated bibliography which will succinctly summarize the study by answering the above questions. Some scientists like to share their annotated bibliographies on personal blogs as a way to engage with the research community and broadcast studies they think are important or interesting. Imagine you are writing annotated bibliographies in the form of blog post for the other students in the class, who are also researching reproductive diversity but most likely on other topics. You can find some excellent examples of annotated bibliographies in blogging style on this site: http://www.joneslabbowdoin.com/new-paper-friday-1

Write your blog post with your peers in mind. What are the essential take-aways? Including images of the animal, graphs of the result, or personal illustrations can help the reader to visualize and help to break up the post so it’s not just a wall of text. While the exact structure of your post is flexible, it will be necessary to have some sort of structure that makes it easy to follow as a reader rather than a list of thoughts and questions.

It's important in these posts to not lose your own voice. Readers of your blog won't just want a summary – they can just read the abstract for that. Practice using the first person. At this stage, when just starting out, it can be especially useful to say explicitly what you did not understand. So, don't be afraid to point this out. Also, don't be afraid to be enthusiastic about something you found especially interesting! Were the results surprising or expected?

Demonstrate that you not only have read the paper but thought about it too. What did you find most interesting or compelling? What are you most curious to learn about next? If you could follow up on this paper with another study, what would you try to observe or what would you want to know? You will come back to these thoughts in future assignments.

Papers Assigned to Students:

- Jukema, J., & Piersma, T. (2006). Permanent female mimics in a lekking shorebird. *Biology Letters*, *2*(2), 161-164. https://doi.org/10.1098/rsbl.2005.0416
- Pietsch, T. W. (1975). Precocious sexual parasitism in the deep sea ceratioid anglerfish, *Cryptopsaras couesi* Gill. *Nature*, *256*(5512), 38-40. https://doi.org/10.1038/256038a0
- Paczolt, K. A., & Jones, A. G. (2010). Post-copulatory sexual selection and sexual conflict in the evolution of male pregnancy. *Nature*, *464*(7287), 401-404. https://doi.org/10.1038/nature08861
- Casselton, L. A. (2002). Mate recognition in fungi. *Heredity*, *88*(2), 142-147. https://doi.org/10.1038/sj.hdy.6800035
- Tobler, M., & Schlupp, I. (2005). Parasites in sexual and asexual mollies (Poecilia, Poeciliidae, Teleostei): a case for the Red Queen?. *Biology letters*, *1*(2), 166-168. https://doi.org/10.1098/rsbl.2005.0305
- Todd, E. V., Ortega-Recalde, O., Liu, H., Lamm, M. S., Rutherford, K. M., Cross, H., ... & Gemmell, N. J. (2019). Stress, novel sex genes, and epigenetic reprogramming orchestrate socially controlled sex change. *Science Advances*, *5*(7), eaaw7006. https://doi.org/10.1126/sciadv.aaw7006

Science Communication: Comparing and Contrasting Popular and Academic Writing Styles

750-900 words

Learning objectives:

- Compare and contrast the writing styles of science papers versus scientific journalism
- Understand what is gained and lost when writing for academic or public audiences
- Learn to make a claim, then back up that claim with evidence from literature
- Be able to quote and refer to others' writing in order to effectively convey a point

After collecting data and analyzing the results, scientists write up their study and submit the paper to a scientific journal. Other scientists who are experts in the same topic are then asked to critique the study. This process, "peer review", allows a journal editor to judge whether a study can be published (note that

the term "peer review" here is used differently than we typically use it in class). The audience of a scientific paper, therefore, are the scientific "peers". Scientific papers are stylistically written with this in mind. The authors must write in a way that incorporates the findings of the research into the broader "conversation" or "debate" on the research topic, which typically occurs between a group of experts who study similar questions.

Once an article is published, science journalists may find these papers and write about the discovery. A journalist wants to convey the same results, but the audience is different. Instead of a conversation with expert peers, a journalist seeks to reach a wider public audience that likely is unaware of the context in which the study occurred. A journalist faces the challenge of achieving this objective while also accurately portraying the study and its results. This process, science communication (or sometimes scicomm), has unique challenges but is an increasingly critical job. This is immediately evident with the Covid-19 pandemic and the global climate change crisis.

Both author types, scientists and journalists, try to convey an answer to the same question: How does this study add to or change what we already know about this topic and why should you, the reader, care? The purpose is the same, but the audience is different.

In this assignment you will find an article written for a public audience on a scientific discovery and compare it with the actual research paper it describes*. This paper should not simply be a summary of the two writing types. Instead, I would like you to evaluate and make a claim on how successfully the journalists convey the science and why. Use quotes and reference both papers throughout in order to back up your claims. Your essay should convey your judgement on the communicative success of both articles, but should also demonstrate that you understand why, from a stylistic and rhetorical perspective, you came to that judgement.

Papers Assigned to Students:

- Fahnestock, J. (1998). Accommodating science: The rhetorical life of scientific facts. *Written Communication*, *15*(3), 330-350. https://doi.org/10.1177/0741088386003003001

*There are a variety of potential ways in which the two articles (academic and public oriented) could be chosen. In this particular course, students were asked to independently find their scientific paper and news article on a similar topic as what they researched in their blogpost assignment (see above in this Supplement). I asked them to send their pair of articles to me, just to make sure the topic was sufficiently related to the course material.

Speculative Fiction: Science as Storytelling

A short, fictional story and an afterword

- Story: 1.5 – 2.5 pages (single-spaced, 12 pt font, 1" margins, a sensible font)
- Afterword: 400-500 words

Learning Objectives:

- Develop and practice your skills as a narrator in both fiction and non-fiction writing

- Use reflection as a tool to identify personal sources of creativity
- Reflect on the ways in which societal and cultural norms are intricately tied to reproductive biology

Authors of speculative fiction have a long history of finding inspiration in the natural world. For instance, Octavia Butler's "Blood Child" and Ursula Le Guin's "Coming of Age in Karhide" both presented an intelligent species with a type of reproductive biology distinctly different from our own. The stories explore some of the implications of that reproductive biology for the society and culture they depict.

In this assignment you will be exploring the social and cultural implications of a nonhuman form of reproductive biology for an advanced society of intelligent, sentient beings. Your story should be based on the research topic you have been studying for the semester. (You can choose another type of reproductive biology, however, you'll need to set up a meeting with me to demonstrate that you have sufficient understanding of the topic).

First, consider how normative reproductive biology of humans may affect various aspects of our societal relationships, culture, art, politics, gender roles, and religious beliefs. *How would these institutions be different in a society with a different form of reproductive biology? What would the life of an individual in this society be like?* **Your response to one of these questions will be the theme of your story**. You might, as in "Blood Child," consider how one species' reproductive biology impacts inter-species relationships. You might imagine another planet and/or time, like Karhide, in which the sex-gender system is far different from that which we experience in our own lives. I encourage you to be creative, but also to make sure that your narrative is grounded in your research on reproductive diversity. To that end, **please make sure that the organism/biological system serving as the basis for your thought experiment is clear through the story itself**. Remember that this is a **short** story, not an anthology on the species or world you have imagined.
What sort of animals do the characters have to be? The characters in your story can take different forms: they could be very humanlike but have the different reproductive method you researched. They could be the same creatures you researched but narrated as if they have a consciousness representable in English in your story. They could be entirely fictional creatures (alien, alternate history, future evolution, etc.) that have this form of reproduction and society surrounding it.

As you write and develop your story, keep in mind these goals: 1) Strive for rich characters that are memorable to the reader. 2) Have a theme, or a take-home message. 3) Carry your reader with a clear sense of movement, or plot, from beginning to end.

The second part of this assignment is an afterword to your story. The afterword to "Blood Child" is an excellent example. Imagine the reader to be someone who knows little of the science behind your research but someone who would be interested in where you drew your inspiration.
Give the reader some detail on the organism you researched, but this should not be the main aspect. Instead, I would like to know about the process in which you shifted from research on the science about a real organism to the imaginative process of writing speculative fiction. This section should be about you

as a writer! This is your moment to reflect on your own writing process and how you draw inspiration. How did you come up with your characters? Did you draw from your own life or others?

Have fun, and I look forward to seeing what you come up with!

*Credit to Dr. Karen Warkentin and Dr. Carrie Preston, WS 101, Boston University, for parts of this prompt

Readings students did before this assignment:

- Butler O. 1995. Bloodchild. In: Bloodchild and Other Stories.
    - Including the afterward
- Le Guin, Ursula K. "Coming of Age in Karhide." The Birthday of the World and Other Stories, HarperCollins, 2002.
    - Including forward
